# Supplementary material for: Activation of LXRβ inhibits tumor respiration and is synthetically lethal with Bcl‐xL inhibition
Source: EMBO Mol Med. 2019 Aug 29;11(10):e10769. doi: 10.15252/emmm.201910769 (PMC6783693; doi:10.15252/emmm.201910769)

Table of Contents

Appendix Figure S1: Enhanced apoptosis induction by the combination treatment of BH3-mimetics and LXR623.

Appendix Figure S2: The LXR agonists sensitize for BH3-mimetic mediated cell death in a predominantly Bcl-xL dependent manner.

Appendix Figure S3: The LXR agonists GW3965 and LXR623 regulate the expression of anti- and pro-apoptotic Bcl-2 family members, favoring an increase in Noxa protein levels and in turn sensitizing for ABT263 mediated apoptosis in a Noxa dependent manner.

Appendix Figure S4: LXR agonists mediate loss of ATP and trigger an unfolded protein response.

Appendix Figure S5: LXR agonists elicit an integrated stress response associated with energy starvation.

Appendix Figure S6: LXR agonists reprogram glutamine and glucose metabolism as shown by carbon tracing analysis.

Appendix Figure S7: LXR agonists suppress oxidative phosphorylation through LXRβ.

Appendix Figure S8: LXR agonist mediated suppression of oxidative phosphorylation is implicated in its induction of cell death.

Appendix Figure S9: Impact of LXRβ on tumor cell respiration of tumor and normal cells.

Appendix Figure S10: Anti-cancer activity of ABT263 and LXR agonists in vivo.

Appendix Figure Legends

Appendix Figure S1. Enhanced apoptosis induction by the combination treatment of BH3-mimetics and LXR623.

A-D, CI (Combination Index) value indicates [as to] whether the drug combination is [either] additive (CI value = 1.0), synergistic (CI value < 1.0) or antagonistic. The dotted line represents additivity with respect to the combination treatment.

E, EC_{50} values based on the dose responses of each drug in the indicated cell cultures.

F, U87 cells were treated with increasing concentration of LXR623 in the presence or absence of cholesterol for 72h. Thereafter, cells were stained with annexin V/propidium iodide and analyzed by multi-parametric flow cytometry.

G, U87 cells were treated with the indicated concentrations of ABT263, LXR623 or the combination of both in the presence or absence of cholesterol for 72h. Thereafter, cellular viability was analyzed and statistical analysis was performed. Shown are means and SD (*n* = 3-4). **p = 0.0039. Statistical significance was determined by two-sided student's t.test.

Appendix Figure S2. The LXR agonists sensitize for BH3-mimetic mediated cell death in a predominantly Bcl-xL dependent manner.

Representative flow plots of melanoma cells, A375 and WC62, treated with ABT263, LXR623 or the combination with subsequent staining for Annexin V/propidium iodide, followed by multi-parametric flow cytometric analysis.

Appendix Figure S3. The LXR agonists GW3965 and LXR623 regulate the expression of anti- and pro-apoptotic Bcl-2 family members, favoring an increase in Noxa protein levels and in turn sensitizing for ABT263 mediated apoptosis in a Noxa dependent manner.

A, T98G, LN229 and MDA-MB-231 breast carcinoma cells were treated with increasing concentrations of GW3965 for the indicated time. Thereafter, whole cell protein lysates were collected and analyzed for the expression of pro- and anti-apoptotic Bcl-2 family members.

B, MeWo melanoma cells were treated with increasing concentrations of LXR623 for the indicated time. Thereafter, whole cell protein lysates were collected and analyzed for the expression of pro- and anti-apoptotic Bcl-2 family members.

C, LN229 cells were transfected and treated as indicated in Figure 3C. Shown are means and SD (n = 3). ****p < 0.001. Statistical significance was determined by two-sided student's t.test.

Appendix Figure S4. LXR agonists mediate loss of ATP and trigger an unfolded protein response.

A, HCT116 cells were treated with 20 μM LXR623 for 24h. Transcriptome and gene set enrichment analysis was performed. Shown are enrichment plots. NES: normalized enrichment score.

B, U87 cells were treated with increasing concentration of LXR623 for 7h. Thereafter, cells were stained with annexin V/propidium iodide and analyzed by multi-parametric flow cytometry.

C, D, HCT116 colonic carcinoma cells were treated with increasing concentrations of GW3965 or LXR623 and analyzed for the levels of ATP after 7h. Shown are means and SD (n = 4). ****p < 0.001. Statistical significance was determined by two-sided student's t.test (C) and one-way ANOVA (D).

E, HCT116 colonic carcinoma cells were treated with increasing concentrations of GW3965 or LXR623 for 7h with subsequent staining for Annexin V/propidium iodide, followed by multi-parametric flow cytometric analysis.

Appendix Figure S5. LXR agonists elicit an integrated stress response associated with energy starvation.

A, B, HCT116 or U87 cells were treated with ABT263, Oligomycin (complex V inhibitor) or the combination and subjected for annexin V/propidium iodide staining, followed by multi-parametric flow cytometry. Shown are means and SD (*n* = 3). ***p = 0.0002, ****p < 0.0001. Statistical significance was determined by one-way ANOVA.

C, HCT116 cells were treated with 20 μM LXR623 for 24h. Transcriptome and gene set enrichment analysis was performed. Shown are enrichment plots. NES: normalized enrichment score.

D, HCT116 cells were treated with increasing concentration of GW3965. Protein lysates were analyzed for GRP78, ATF4 and ATF3 by capillary electrophoresis. Vinculin was used as a loading control.

E, U87 cells were treated with DMSO or 20 μM LXR623. RNA was harvested, transcribed to cDNA and cDNA was analyzed for the indicated ER-stress related markers.

F, G, HCT116 cells were treated with GW3965 or LXR623 for 24h. RNA was harvested, transcribed to cDNA and cDNA was analyzed for the indicated ER-stress related markers.

H, U87 or HCT116 cells were treated with oligomycin and whole cell protein lysates were analyzed for the expression of phosphorylated AMPK (threonine 172) and total AMPK, using capillary electrophoresis.

I, HCT116 or U87 cells were treated with oligomycin and whole cell protein lysates were analyzed for GRP78, ATF4, ATF3 and Noxa by capillary electrophoresis.

J, K, HCT116 and LN229 cells were transfected with non-targeting ATF3 or ATF4 siRNAs. After transfection, cells were subjected to treatment with 20 μM LXR623. Whole cell protein lysates were collected and analyzed by standard western blotting for the expression of ATF3, ATF4, Noxa and Actin.

Appendix Figure S6. LXR agonists reprogram glutamine and glucose metabolism as shown by carbon tracing analysis.

A-E, U87 cells were incubated in DMEM (devoid of phenol red, glucose, pyruvate and glutamine) supplemented with 25 mM U-^{13}C-glucose, 4 mM glutamine and 1.5% dialyzed FBS in the presence or absence of 20 μM LXR623 for 24h. Cells were then harvested for LC/MS analysis. The fractions of each different isotopologues of each metabolite were calculated (percentage of the entire pool).

F, The carbon tracing map is included to ease interpretation of the findings. U-^{13}C-derived glutamine carbons (red) can either be directed into oxidative glutamine metabolism (carbons shown in black) or into reductive carboxylation (highlighted in blue). The fractions of each different isotopologues of each metabolite were calculated (percentage of the entire pool).

G-O, U87 cells were incubated in DMEM (devoid of phenol red, glucose, pyruvate and glutamine) supplemented with 4 mM U-^{13}C-Glutamine, 25 mM glucose and 1.5% dialyzed FBS in the presence or absence of 20 μM LXR623 for 24h. Cells were then harvested for LC/MS analysis.

P, U87 cells were treated with 20 μM LXR623 in the presence or absence of glutamine for 48h (media conditions: 25 mM glucose, 4 mM glutamine, 1.5% dialyzed FBS; glutamine deprived media conditions: 25 mM glucose, 1.5% dialyzed FBS). Glut: glutamine. A viability assay

(CyQUANT assay) was performed. Shown are means and SD (*n* = 4). *p = 0.0377. Statistical significance was determined by two-sided student's t.test.

Q, LC-MS analysis from U87 GBM cells treated with 20 μM LXR623 for 24h. Shown are the fold changes (log2) of amino acids (LXR623 vs DMSO).

Appendix Figure S7. LXR agonists suppress oxidative phosphorylation through LXRβ.

A-C, NCH644 GBM stem like cells were treated with increasing concentration of LXR623 for 7h and analyzed for oxygen consumption rate (OCR) on the seahorse analyzer. Oligomycin (OM), FCCP and rotenone/antimycin (R/A) were added in a time sequential manner. From this assay, mitochondrial OCR and coupled respiration were calculated. Shown are means and SD (*n* = 4). ***p = 0.0002 (DMSO vs LXR623 10μM; DMSO vs LXR623 40μM); ***p = 0.0003 (DMSO vs LXR623 20μM); ****p<0.0001. Statistical significance was determined by one-way ANOVA.

D-I, U87 cells were treated with 20 μM LXR623 for 7h or 24h and analyzed for oxygen consumption rate (OCR) on the seahorse analyzer. From this assay, mitochondrial OCR and coupled respiration were calculated. Shown are means and SD (*n* = 3). Statistical significance was determined by two-sided student's t.test.

J-L, LN229 cells were treated with 5 μM LXR623 for 24h and analyzed for the oxygen consumption rate (OCR) on the seahorse analyzer. From this assay, mitochondrial OCR and coupled respiration were calculated. Shown are means and SD (*n* = 5). Statistical significance was determined by two-sided student's t.test.

M-O, HCT116 cells were with LXR623 or GW3965. Thereafter, cells were subjected to extracellular flux analysis (mitochondrial stress assay). Shown are means and SD (*n* = 4-8). ***p = 0.0002, ****p < 0.0001. Statistical significance was determined by one-way ANOVA.

Appendix Figure S8. LXR agonist mediated suppression of oxidative phosphorylation is implicated in its induction of cell death.

A, U87 cells were treated with increasing concentration of LXR623 with low (0.5 mM Glucose) and high glucose (25 mM Glucose) level for 72h. Thereafter, cells were stained with annexin V/propidium iodide and analyzed by multi-parametric flow cytometry.

B, Cellular viability was analyzed in samples treated with increasing concentration of LXR623 with low and high glucose level. Shown are means and SD (n = 3). *p = 0.0115, ***p = 0.0009. Statistical significance was determined by two-sided student's t.test.

C, U87 cells were cultured in the media containing 25 mM Galactose for 7 days and treated with 20 μM LXR623. Thereafter, cellular viability was analyzed and statistical analysis was performed. Shown are means and SD (n = 4). ***p = 0.0005. Statistical significance was determined by two-sided student's t.test.

D, U87 were treated increasing concentrations of LXR623 in the presence or absence of exogenous ATP. Thereafter, cellular viability was analyzed and statistical analysis was performed.

E, U87 were treated increasing concentrations of LXR623 in the presence or absence of 10 mM Pyruvate. Thereafter, cells were stained with annexin V/propidium iodide and analyzed by multi-parametric flow cytometry. Cellular viability was analyzed and statistical analysis was performed. Pyr: Pyruvate.

F, U87 were treated increasing concentrations of LXR623 in the presence or absence of 20 μM Aspartate. Thereafter, cells were stained with annexin V/propidium iodide and analyzed by multi-parametric flow cytometry. Cellular viability was analyzed and statistical analysis was performed. Asp: Aspartate.

G, H, HCT116 cells were treated with increasing concentration of LXR623 or GW3965. Thereafter, whole cell protein lysates were collected and analyzed the protein levels of the respiratory complexes (OXPHOS).

I, NCH644 GBM stem like cells were treated with 20 μM LXR623 or 24h. Thereafter, whole cell protein lysates were collected and analyzed for the protein levels of the respiratory complexes.

Appendix Figure S9. Impact of LXRβ on tumor cell respiration of tumor and normal cells.

A, B, HCT116 cells were transduced with an empty vector or a NR1H2 (encodes for LXRβ) overexpressing construct (LXRβ). The construct contained an HA-tag. Capillary electrophoresis was performed to confirm proper expression. Oxygen consumption rate (OCR) was assessed by the seahorse analyzer. Oligomycin, FCCP and rotenone/antimycine were added in a time sequential manner.

C, D, HCT116 cells were transduced with shRNA targeting NR1H2 (LXRβ) or a non-targeting lentiviral construct. Silencing was confirmed by capillary electrophoresis. Oxygen consumption rate (OCR) was assessed by the seahorse analyzer.

E-G, HCT116 cells were transfected with non-targeting or LXRβ specific siRNA and treated with 2.5 μM LXR623. Silencing was confirmed by capillary electrophoresis. Oxygen consumption rate (OCR) was assessed by the seahorse analyzer. Basal and coupled respiration were calculated. Shown are means and SD (n = 3). *p = 0.0376, ***p = 0.0003. Statistical significance was determined by two-sided student's t.test.

H, I, Human astrocytes were treated with LXR623 or GW3965 for 24h. Oxygen consumption rate (OCR) was assessed by the seahorse analyzer.

J, K, U87 and HCT116 cells were treated with LXR623 or GW3965 for 24h. Cells were starved, the glycolysis stress assay (subsequent injection of glucose, oligomycin followed by 2-DG) was

performed and extracellular acidification rate (ECAR) readings were obtained. Glycolysis was calculated.

Appendix Figure S10. Anti-cancer activity of ABT263 and LXR agonists in vivo.

A-C, Gross images of the in vivo xenograft experiments described in main Figure 7 and method section.

Appendix Figure S1

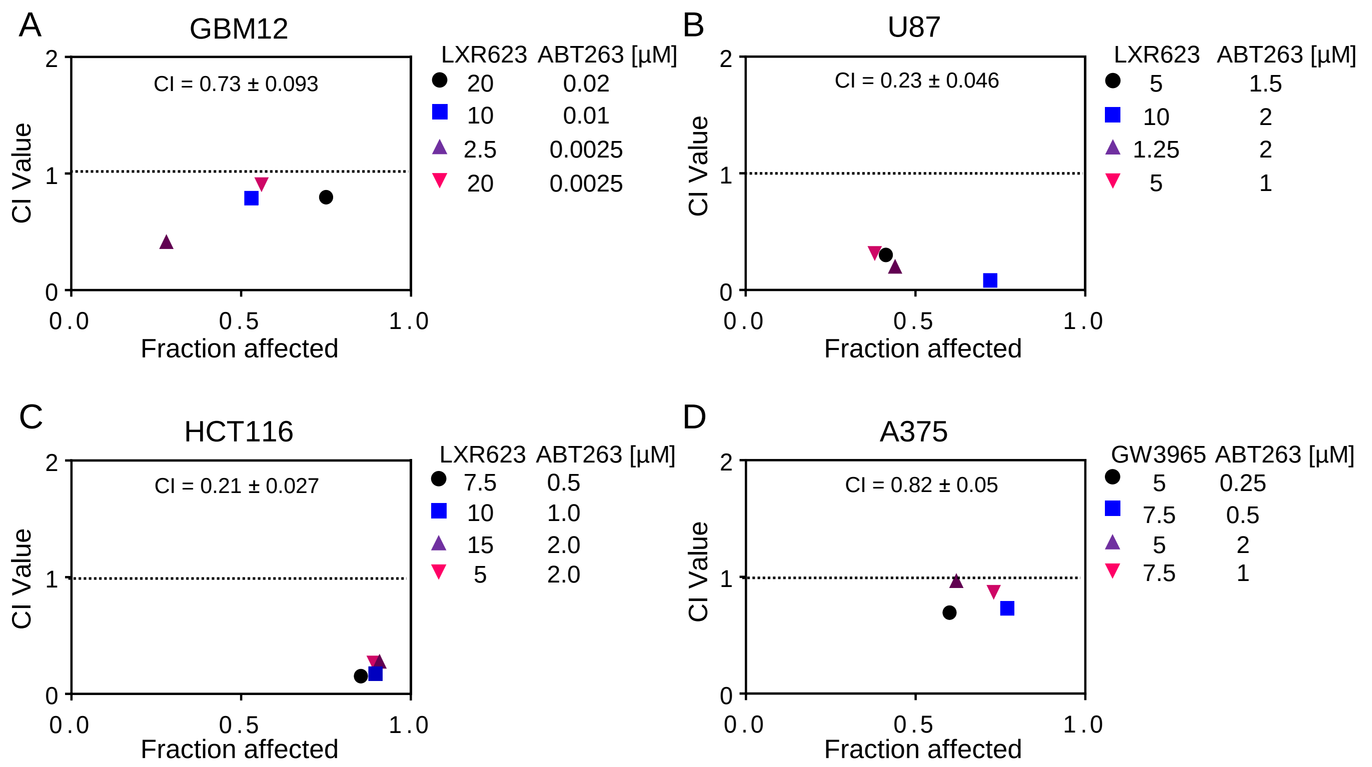

E

EC50		
Cell line	LXR623 (µM)	ABT263 (µM)
GBM12	20.23	0.027
U87	60.85	14.75
HCT116	13.53	1.39

EC50		
Cell line	GW3965 (µM)	ABT263 (µM)
A375	6.40	4.27

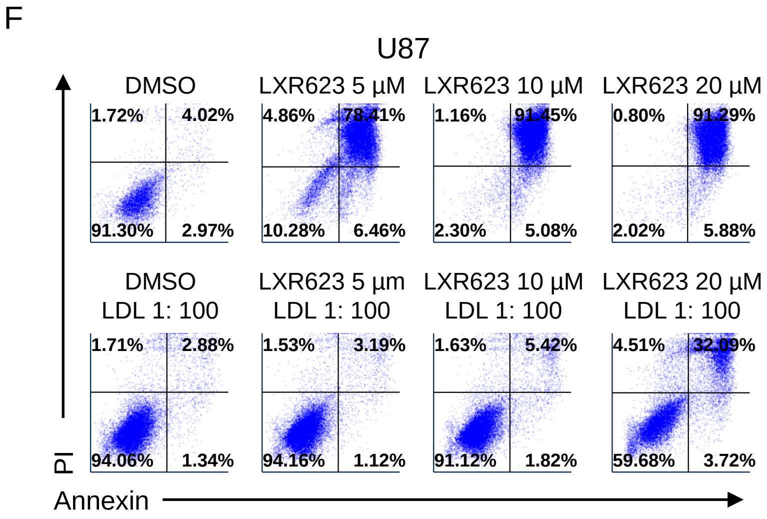

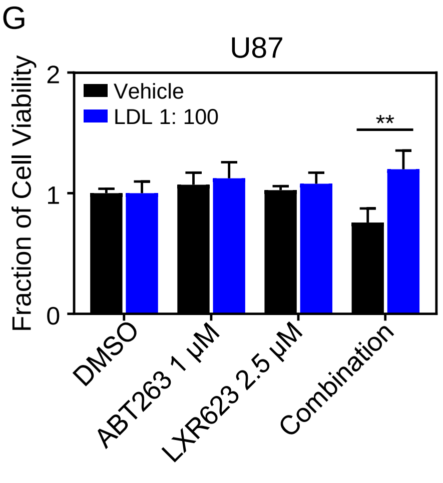

Appendix Figure S2

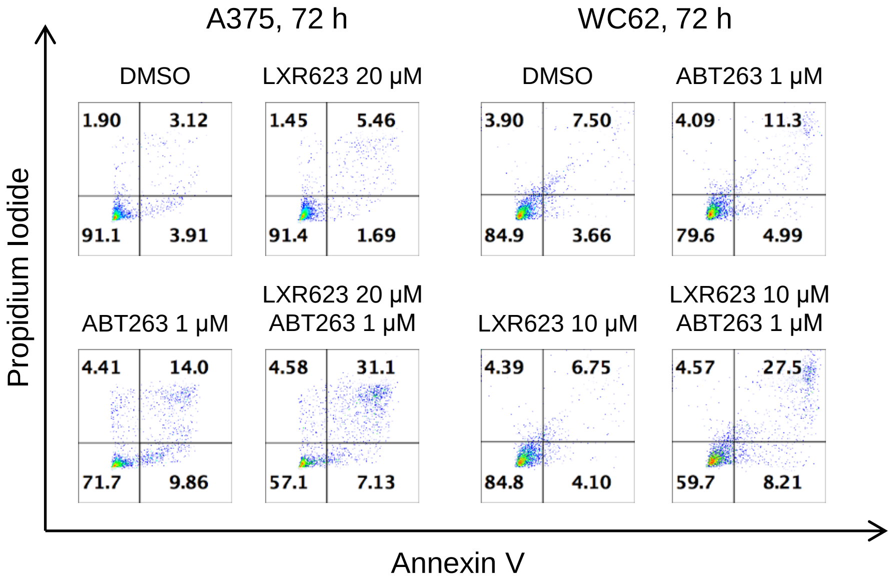

Appendix Figure S3

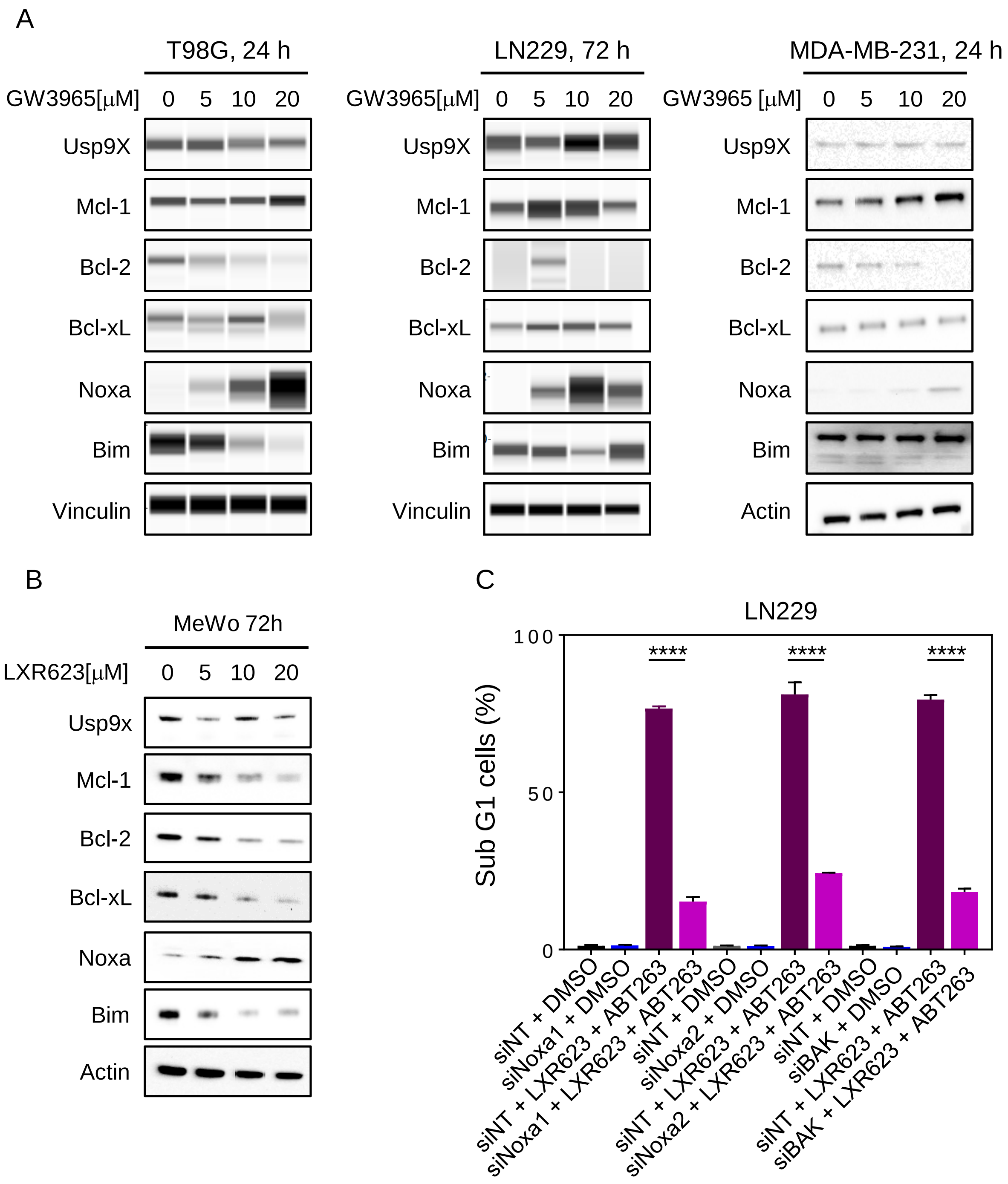

Appendix Figure S4

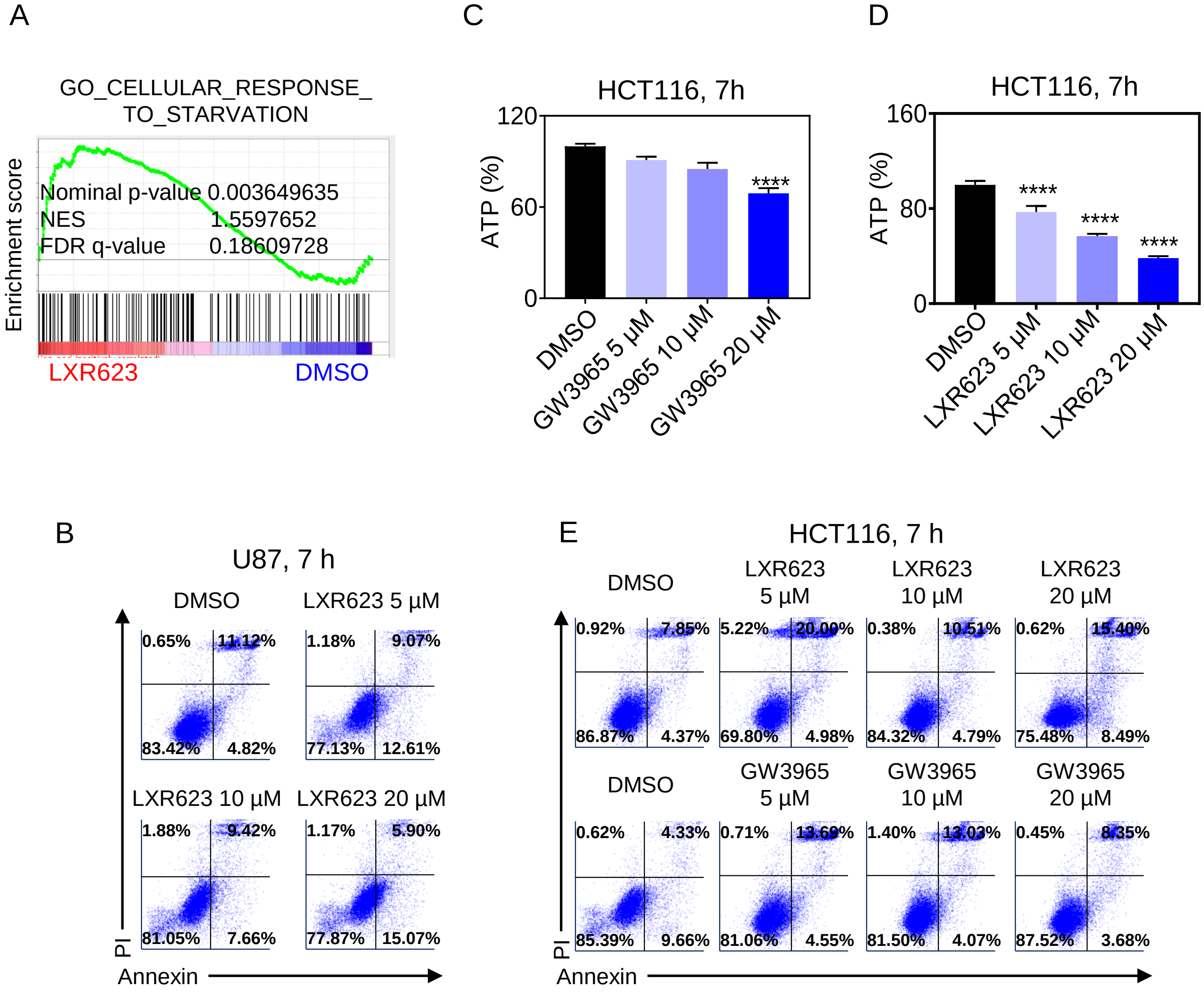

Appendix Figure S5

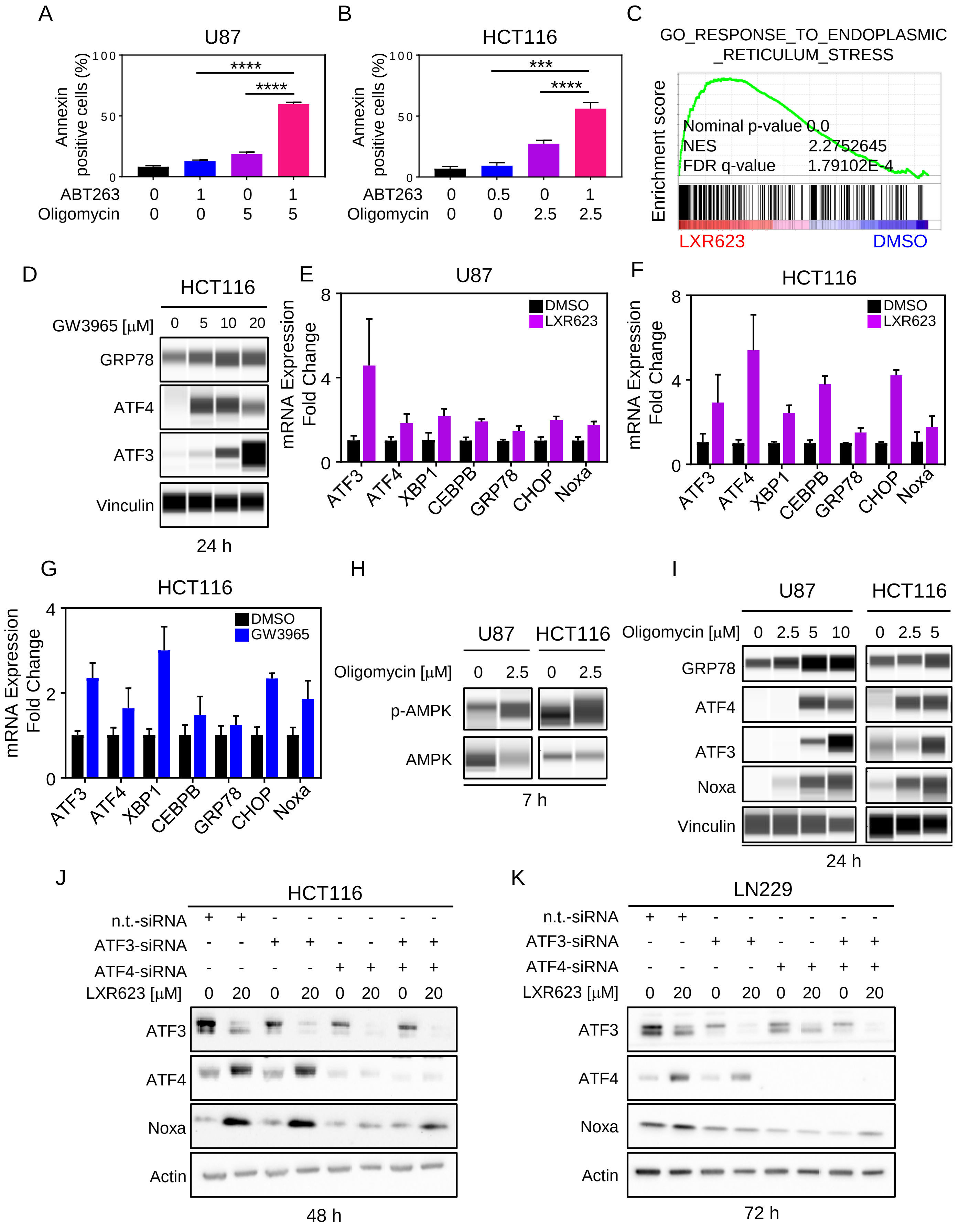

Appendix Figure S6

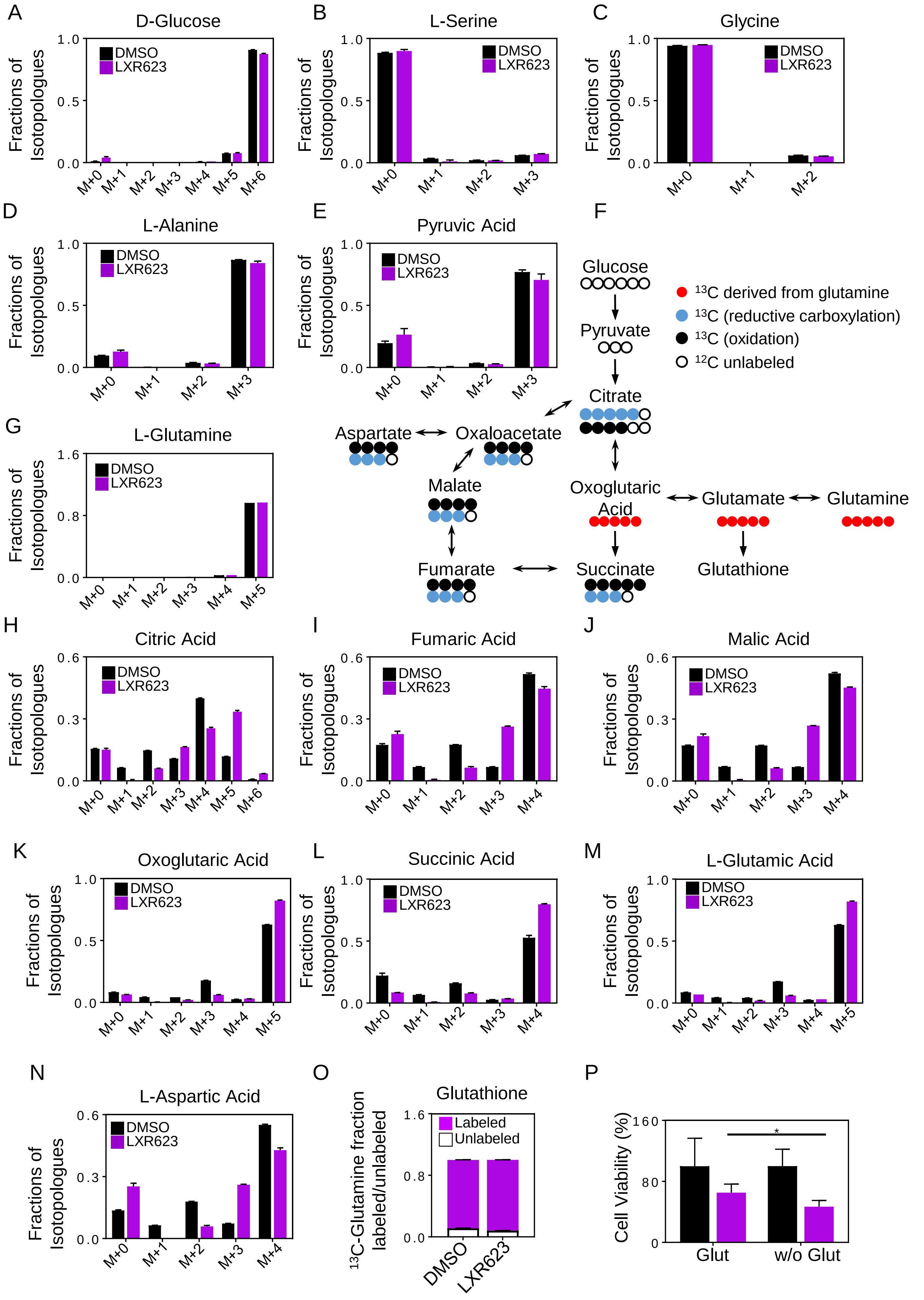

Appendix Figure S6, continued

Q

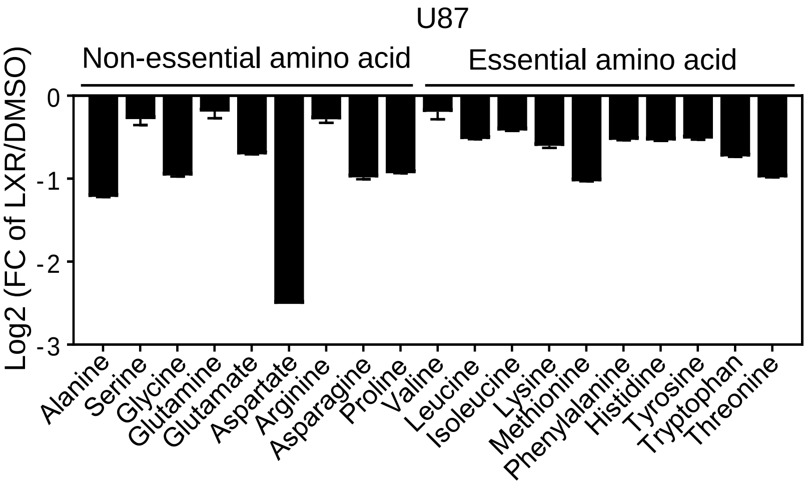

Appendix Figure S7

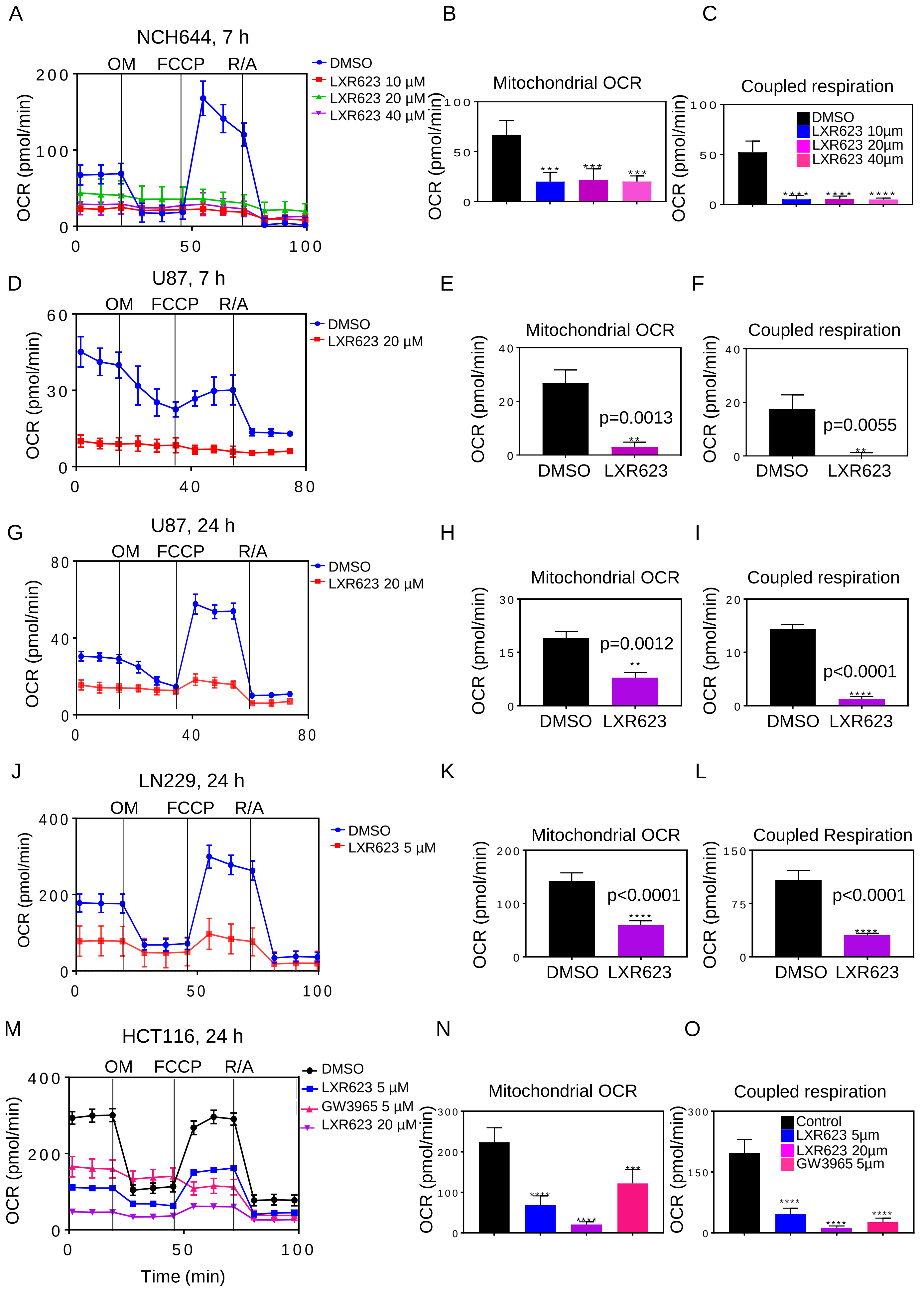

Appendix Figure S8

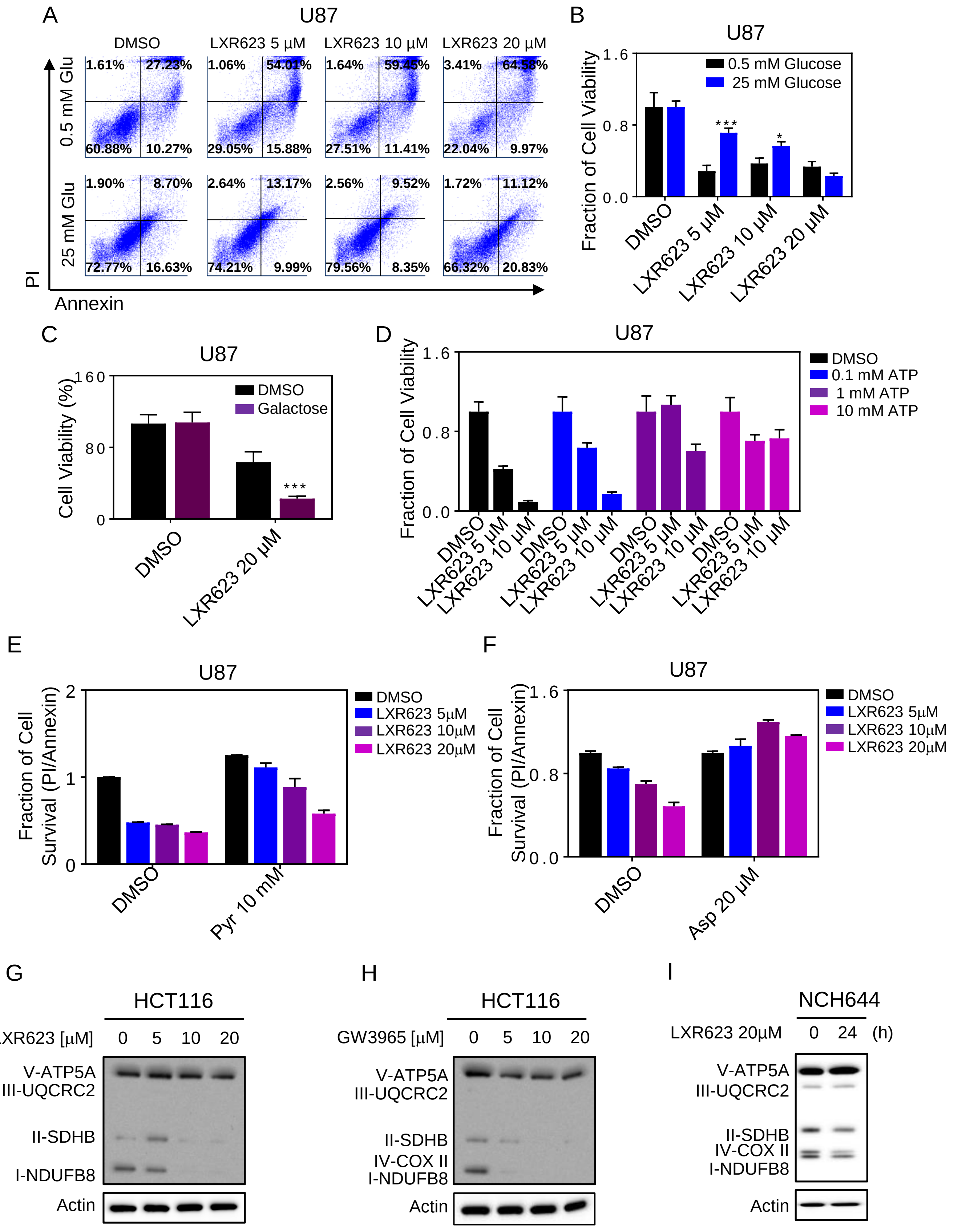

Appendix Figure S9

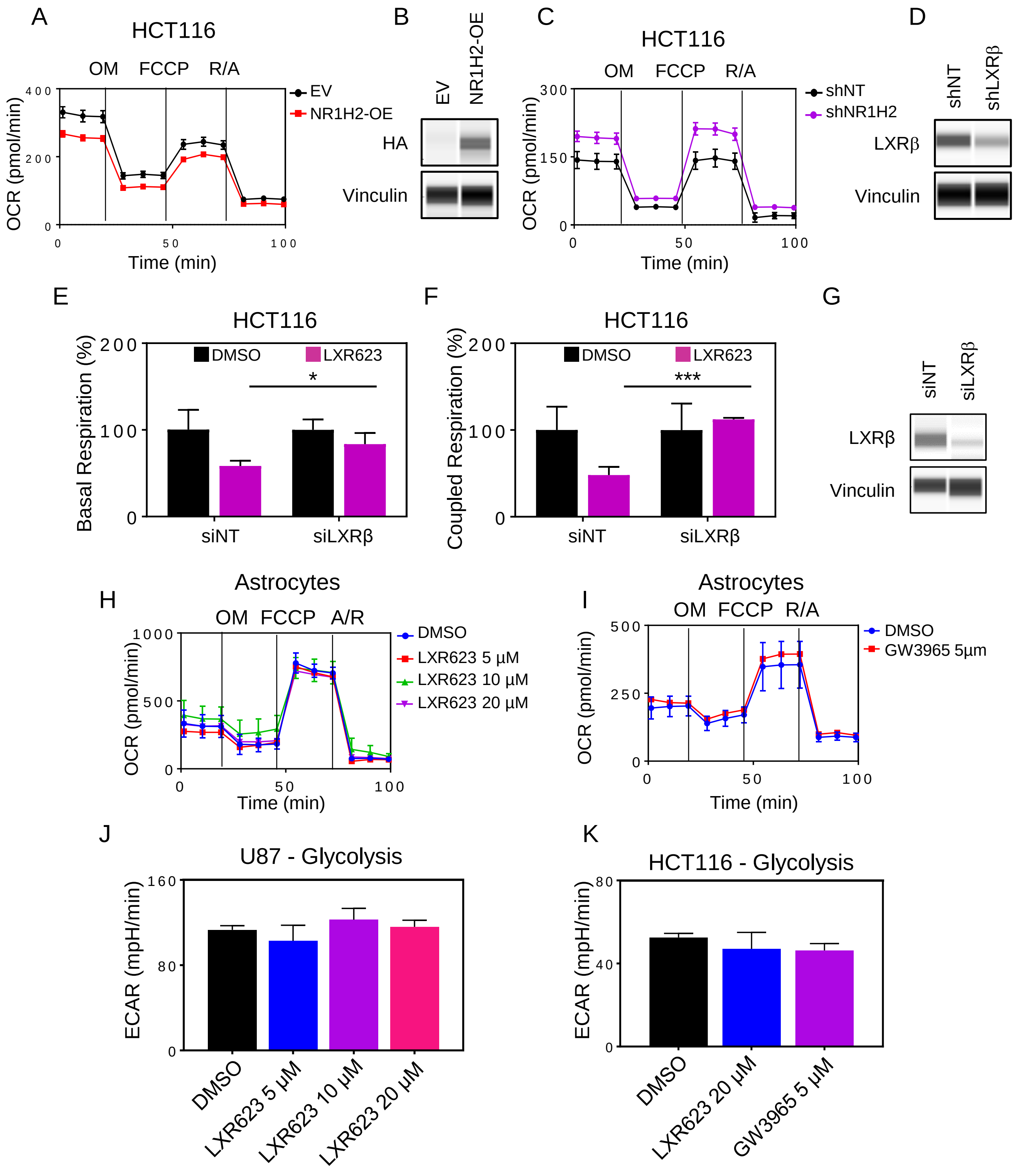

Appendix Figure S10

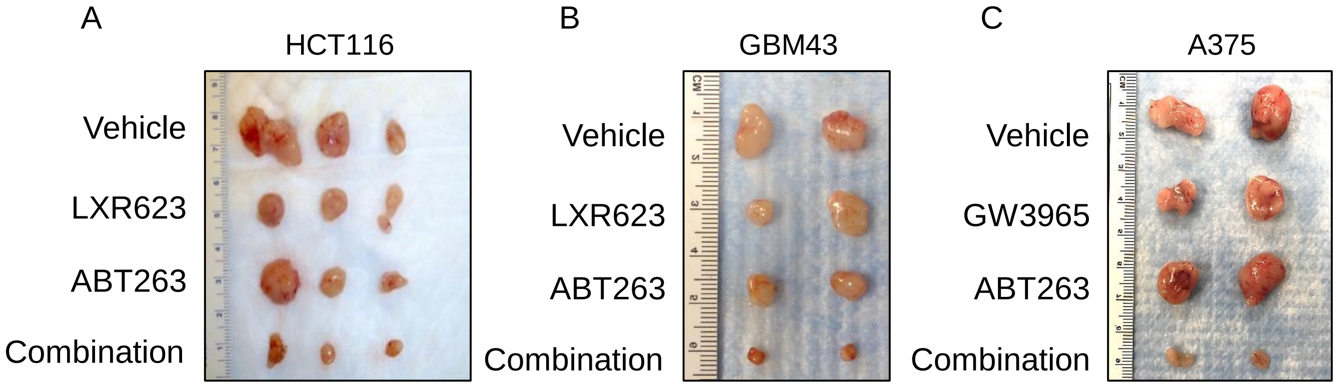

Supplement: Supplementary file 1 — Appendix [file EMMM-11-e10769-s001.pdf]
