# Supplementary material for: Activation of LXRβ inhibits tumor respiration and is synthetically lethal with Bcl‐xL inhibition
Source: EMBO Mol Med. 2019 Aug 29;11(10):e10769. doi: 10.15252/emmm.201910769 (PMC6783693; doi:10.15252/emmm.201910769)
Supplement: Supplementary file 2 — Expanded View Figures PDF [file EMMM-11-e10769-s002.pdf]

Expanded View Figures

Figure EV1. A genetic cholesterol efflux signature correlates with low levels of genes related to mitochondrial metabolism in patients.

A, B HCT116 cells were treated with 20 μM LXR623 for 24 h. Transcriptome and gene set enrichment analysis was performed. Shown are enrichment plots. NES: normalized enrichment score.

C U87 cells were treated with increasing concentrations of LXR623 for 24 h. Thereafter, whole-cell protein lysates were collected and analyzed by capillary electrophoresis for the expression of the ABCA1 transporter. Vinculin was used as a loading control.

D–F HCT116 cells were treated with 20 μM LXR623 for 24 h. Transcriptome and gene set enrichment analysis was performed. Shown are enrichment plots. NES: normalized enrichment score.

G Representative genes from the GSEA derived in (D) (mitochondrial transcription) were interrogated in patients harboring high levels of ABCA1 (> EXP1.5) vs. low levels from the TCGA database (colonic adenocarcinoma). Middle lines in boxplot: median; box ranges: upper: 75th percentile; lower: 25th percentile; error bars: 10th and 90th percentile; number of replicates: n = 382; statistical analysis was determined by two-sided t-test.

H Representative genes from the GSEA derived in (E) (mitochondrial translation) were interrogated in patients harboring high levels of ABCA1 (> EXP1.5) vs. low levels from the TCGA database (colonic adenocarcinoma). Middle lines in boxplot: median; box ranges: upper: 75th percentile; lower: 25th percentile; error bars: 10th and 90th percentile; number of replicates: n = 382; statistical analysis was determined by two-sided t-test.

I Representative genes from the GSEA derived in (F) (mitochondrial protein complex) were interrogated in patients harboring high levels of ABCA1 (> EXP1.5) vs. low levels from the TCGA database (colonic adenocarcinoma). Middle lines in boxplot: median; box ranges: upper: 75th percentile; lower: 25th percentile; error bars: 10th and 90th percentile; number of replicates: n = 382; statistical analysis was determined by two-sided t-test.

J, K Correlation of ABCA1 levels vs. genes involved in electron transport chain (COX5B, UQCC2) and mitochondrial translation in patients with colonic adenocarcinoma (TCGA) or glioblastoma (GBM)(TCGA). Two sided t-test was used for the statistical analysis.

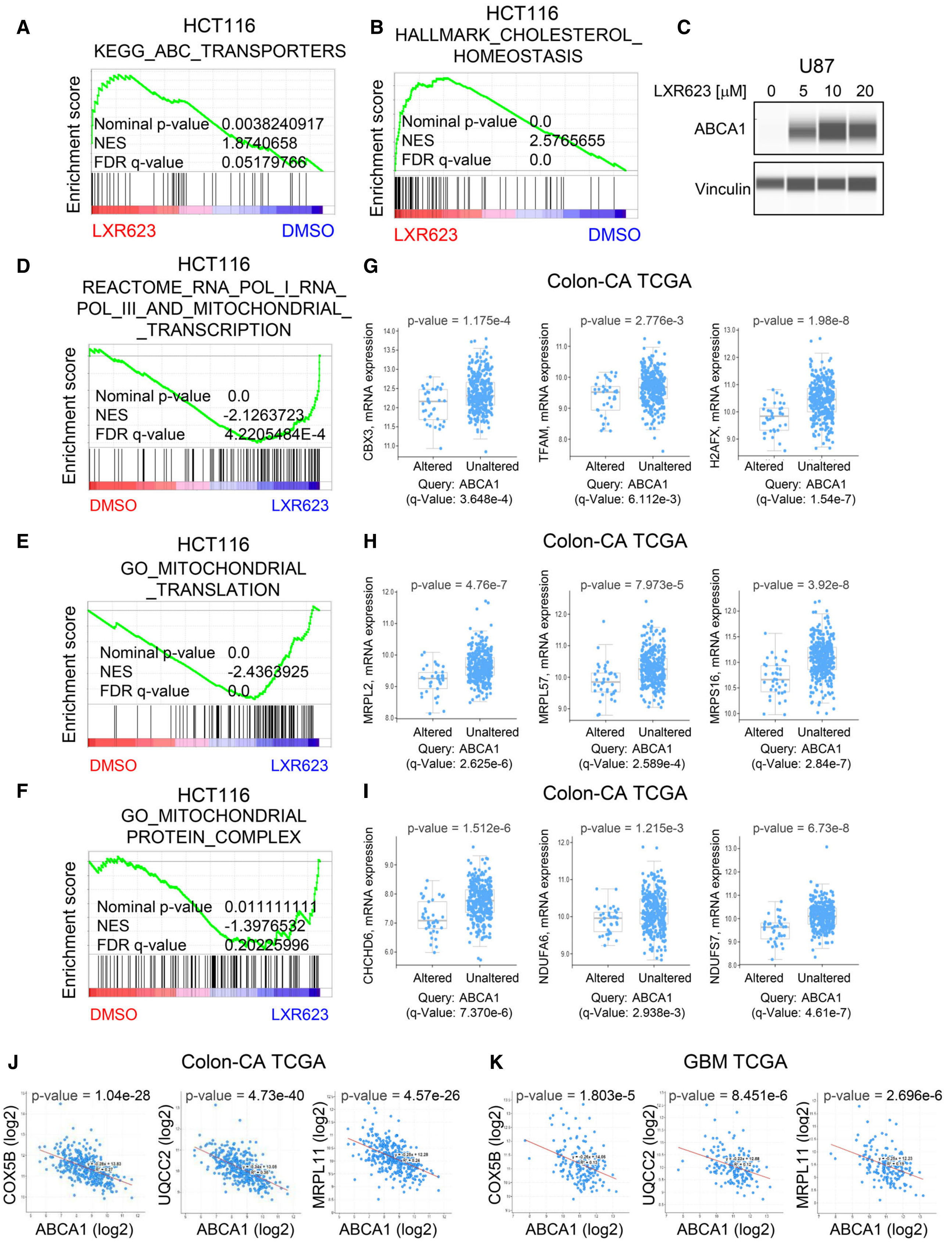

Figure EV1.

© 2019 The Authors

Figure EV2. Volcano plot of patients with high mRNA levels of ABCA vs. low levels and pathway analysis of most significant down-regulated genes in patients with high levels of ABCA1.

A Shown is a volcano plot of patients from the TCGA (database) with high vs. low levels of ABCA1. Highlighted is the mRNA level of ABCA1. In addition, transcripts related to the electron transport chain (ETC) are shown in red and are encircled. A close-up of the down-regulated genes is integrated in main Fig 1F. fc: fold changes; p: *P*-value.

B KEGG pathway analysis was performed on the seventy-five most significant down-regulated genes from the analysis shown in (A). The different KEGG pathways (most significant ones) are shown from the highest to lowest significant changes ($-\log_{10}$ (*P*-value)). Several of the enriched pathways are directly related to mitochondrial metabolism, which is highlighted in red.

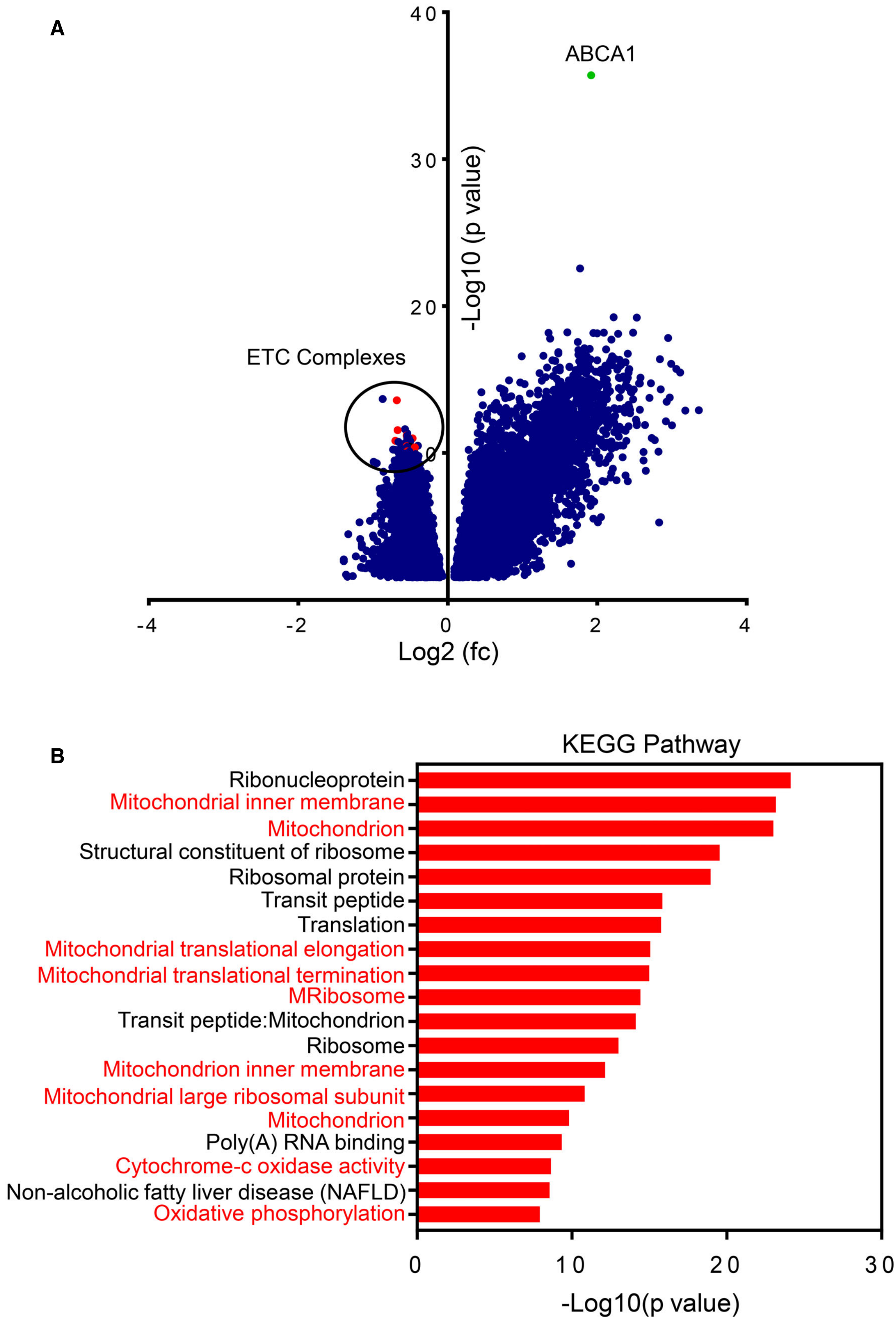

Figure EV2.

 © 2019 The Authors

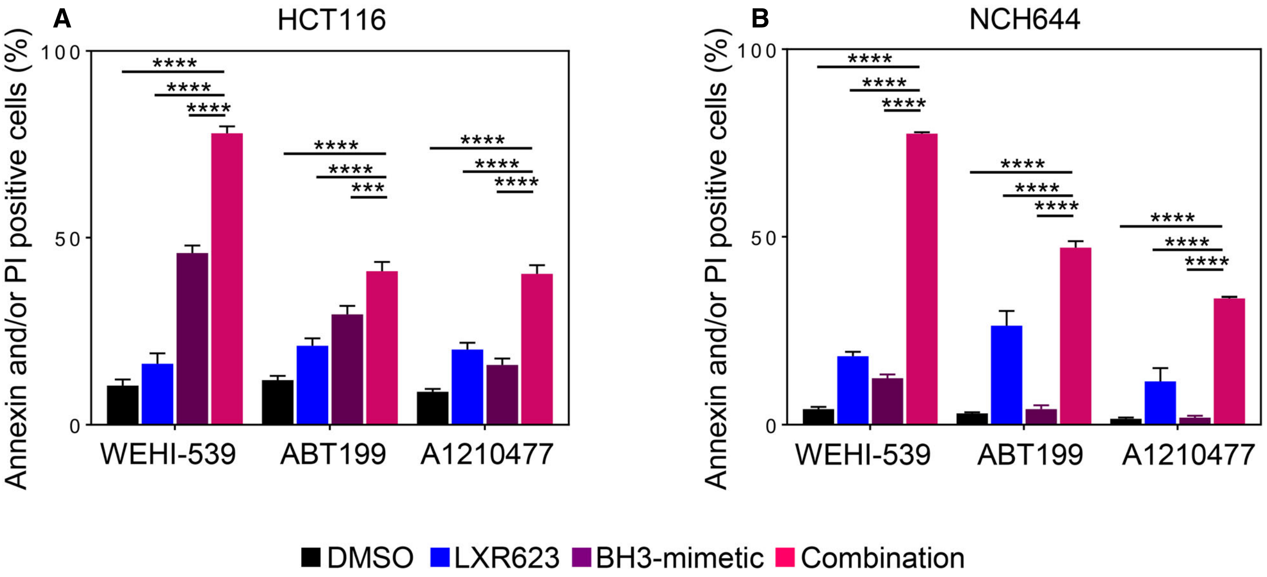

Figure EV3. Quantifications of cell cultures treated with BH3 mimetics, LXR623, or their combination treatment.

A, B HCT116 colonic carcinoma or NCH644 glioblastoma stem cells were treated with selective BH3 mimetics, WEHI-539 (Bcl-xL inhibitor), ABT199 (Bcl-2 inhibitor), or A1210477 (Mcl-1 inhibitor) in the presence or absence of LXR623 for 48 h. Thereafter, cells were labeled with annexin V/propidium iodide and analyzed by multiparametric flow cytometry. Shown are means and SD (n = 3). ***P = 0.0006, ****P < 0.0001. Statistical significance was determined by one-way ANOVA.
