# Supplementary material for: Activation of LXRβ inhibits tumor respiration and is synthetically lethal with Bcl‐xL inhibition
Source: EMBO Mol Med. 2019 Aug 29;11(10):e10769. doi: 10.15252/emmm.201910769 (PMC6783693; doi:10.15252/emmm.201910769)

Source Data Figure S3A-1

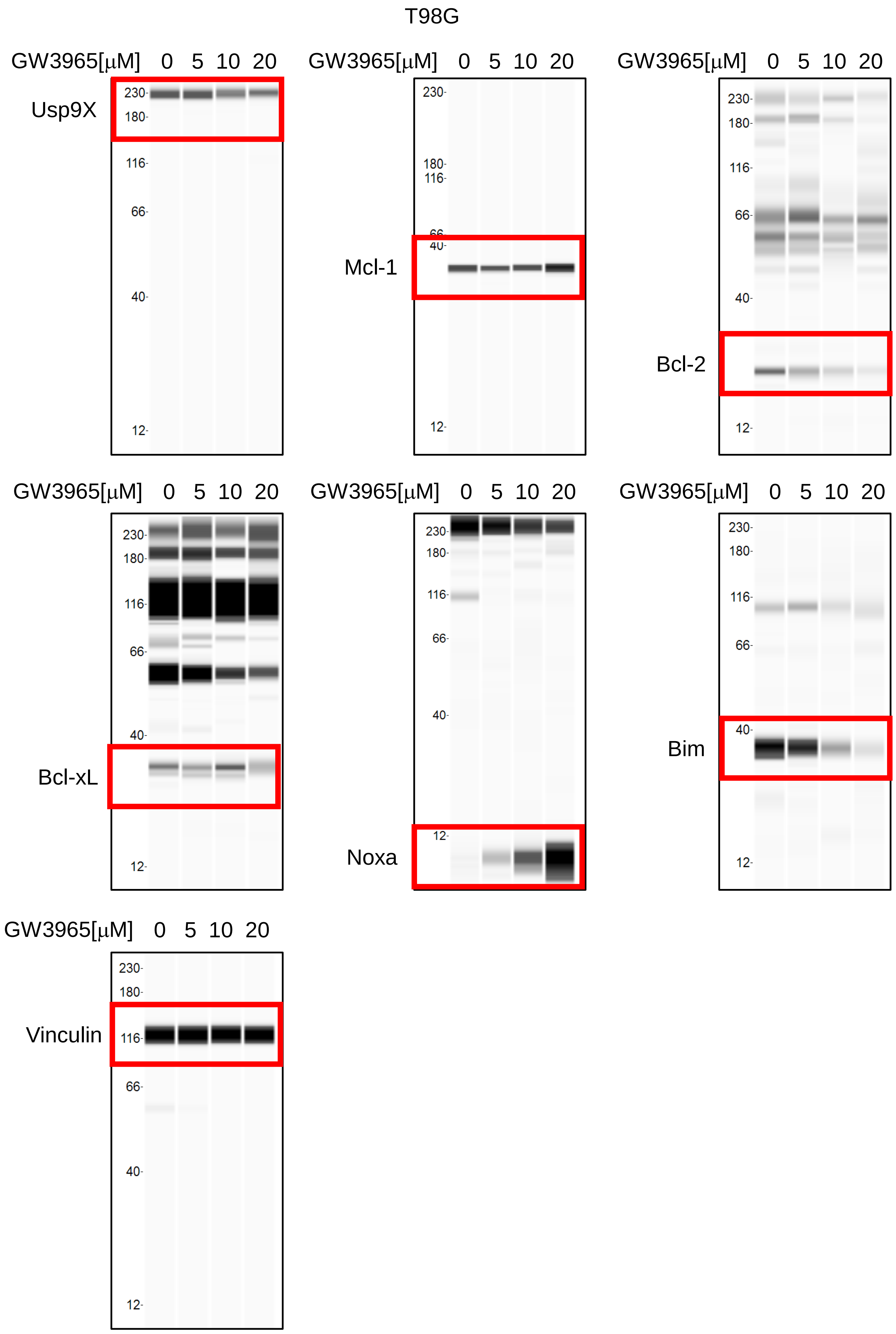

Source Data Figure S3A-2

LN229

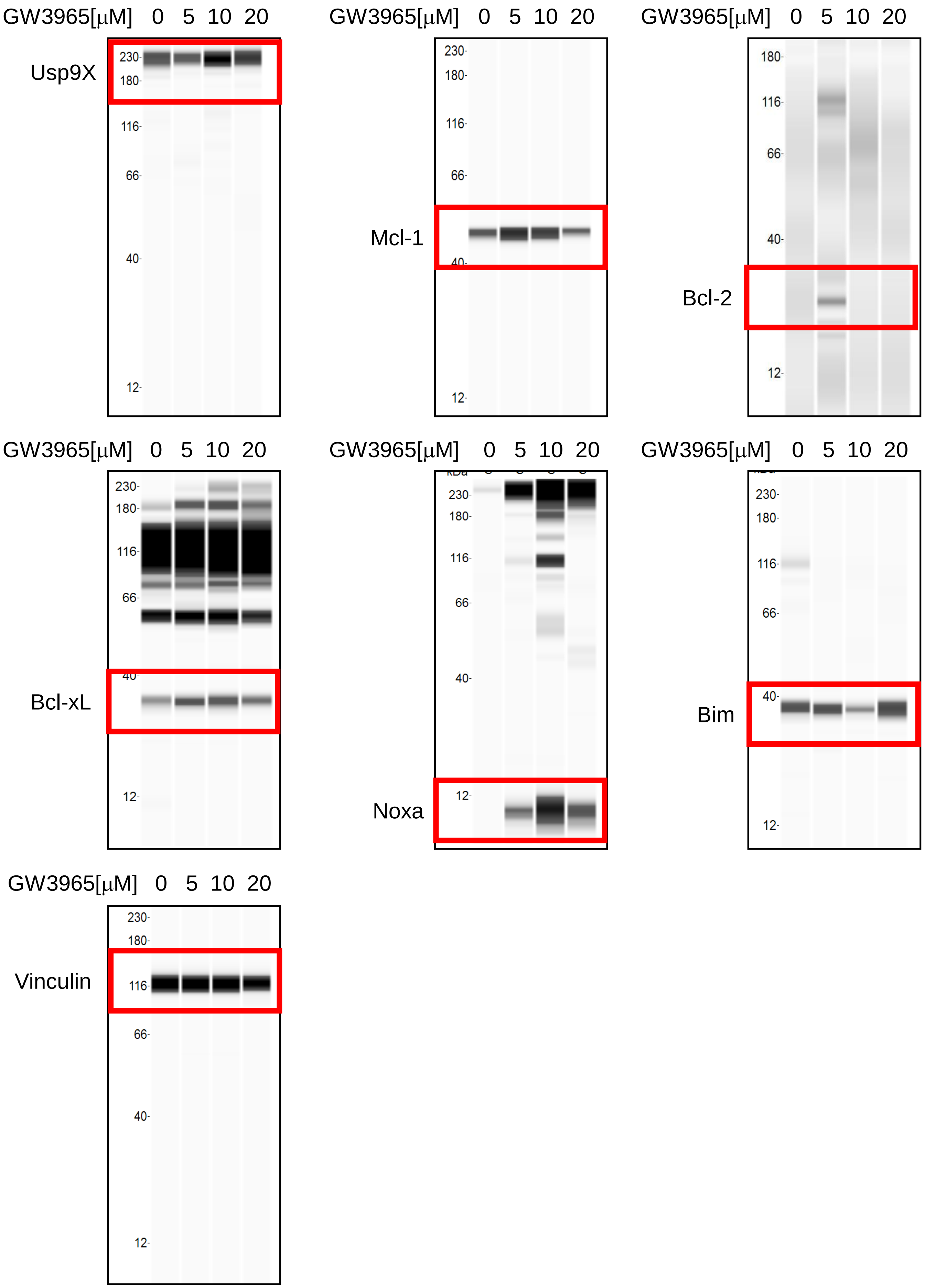

Source Data Figure S3A-3

MDA-MB-231

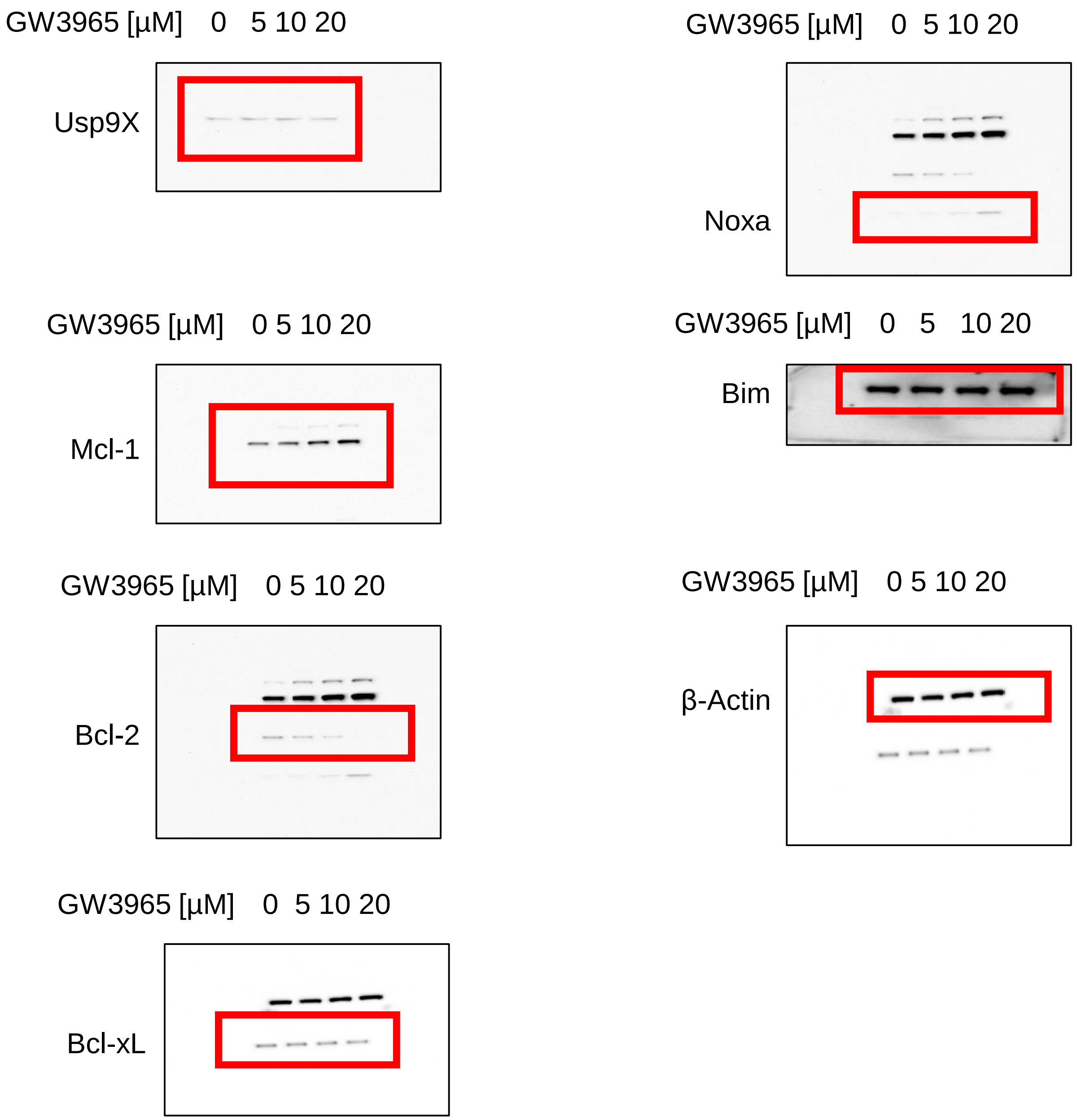

Source Data Figure S3B

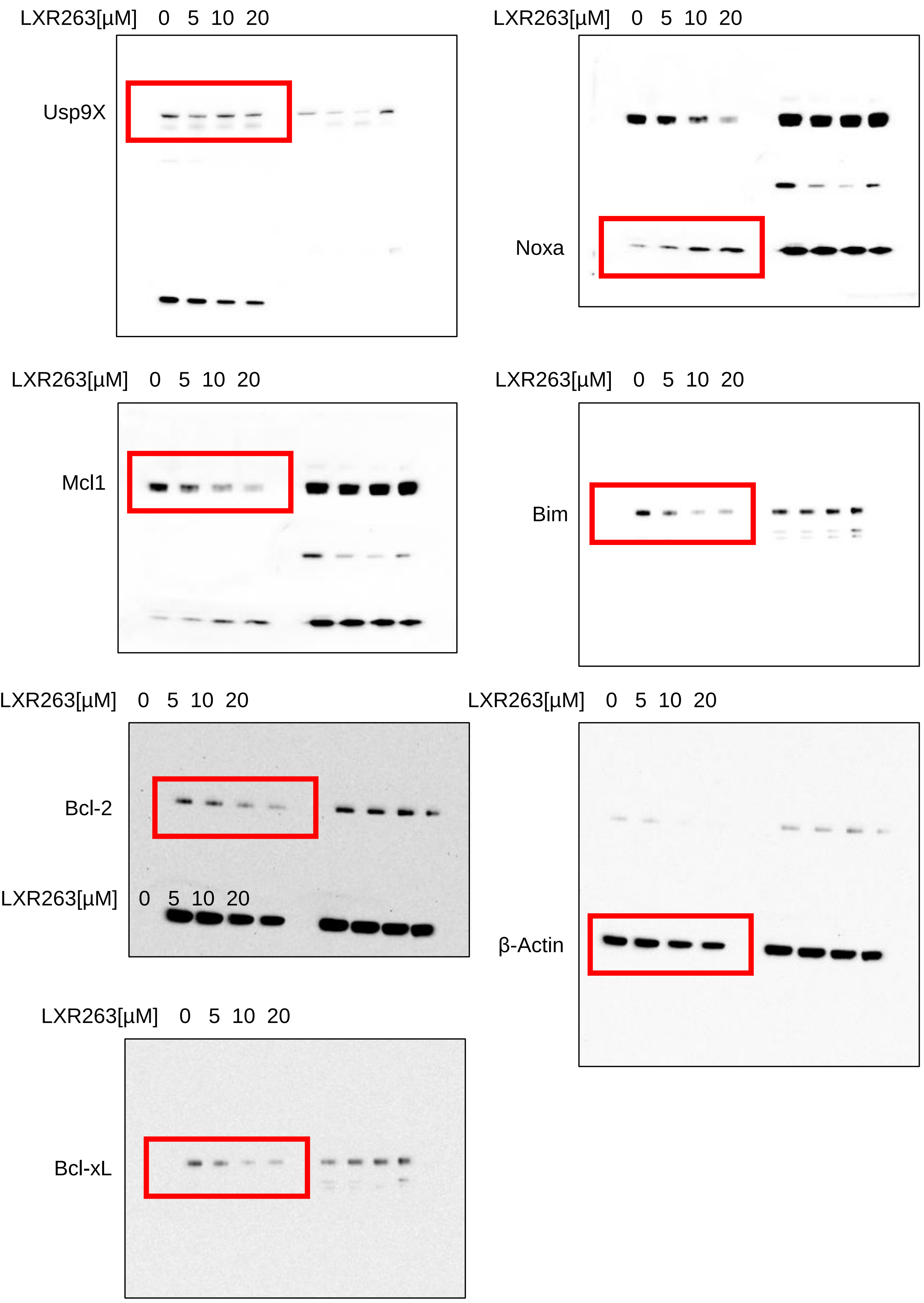

Source Data Figure S5D

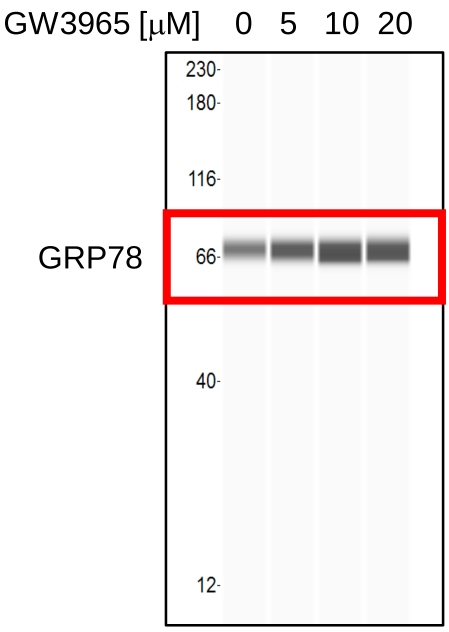

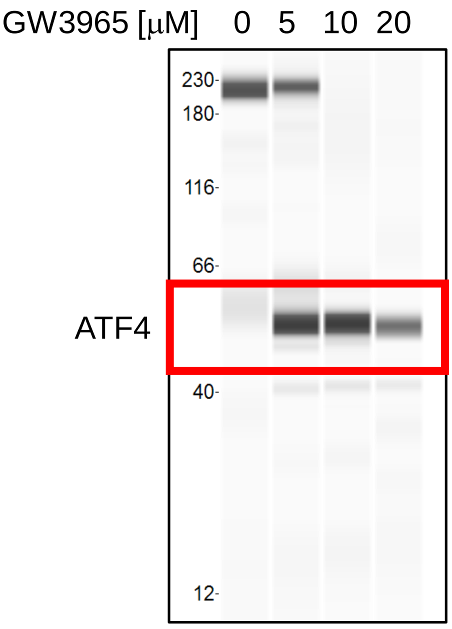

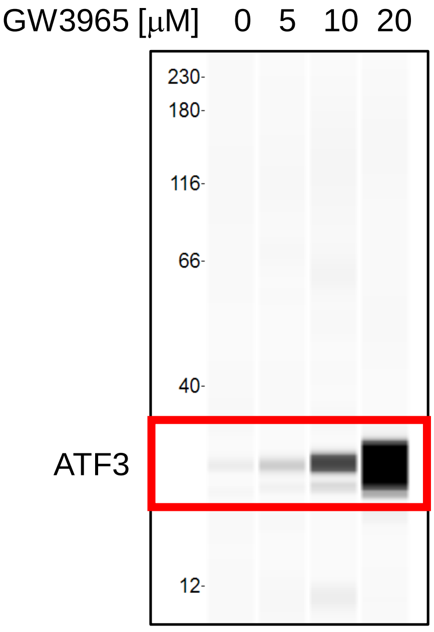

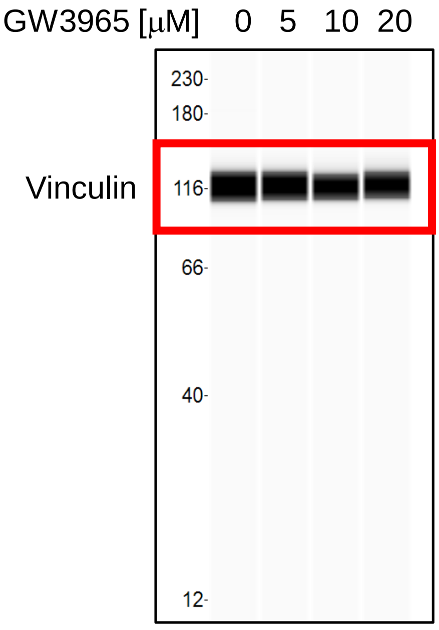

Source Data Figure S5H

U87

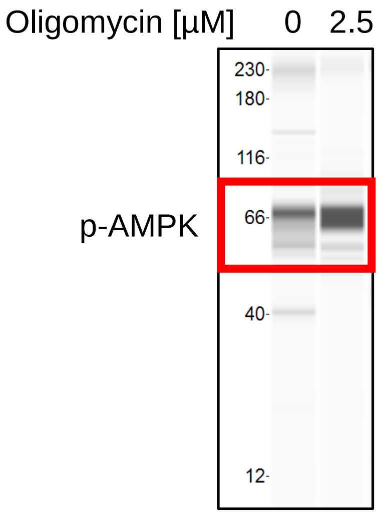

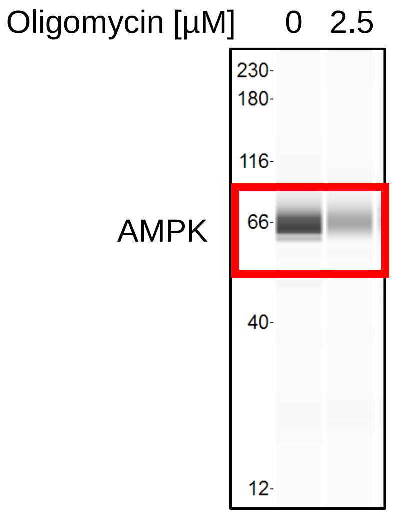

HCT116

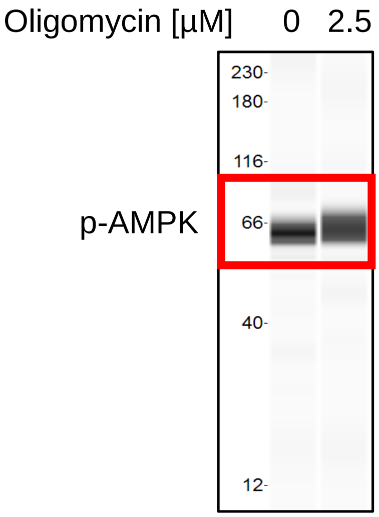

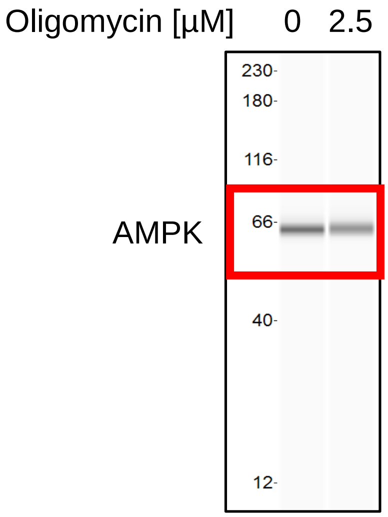

Source Data Figure S5I

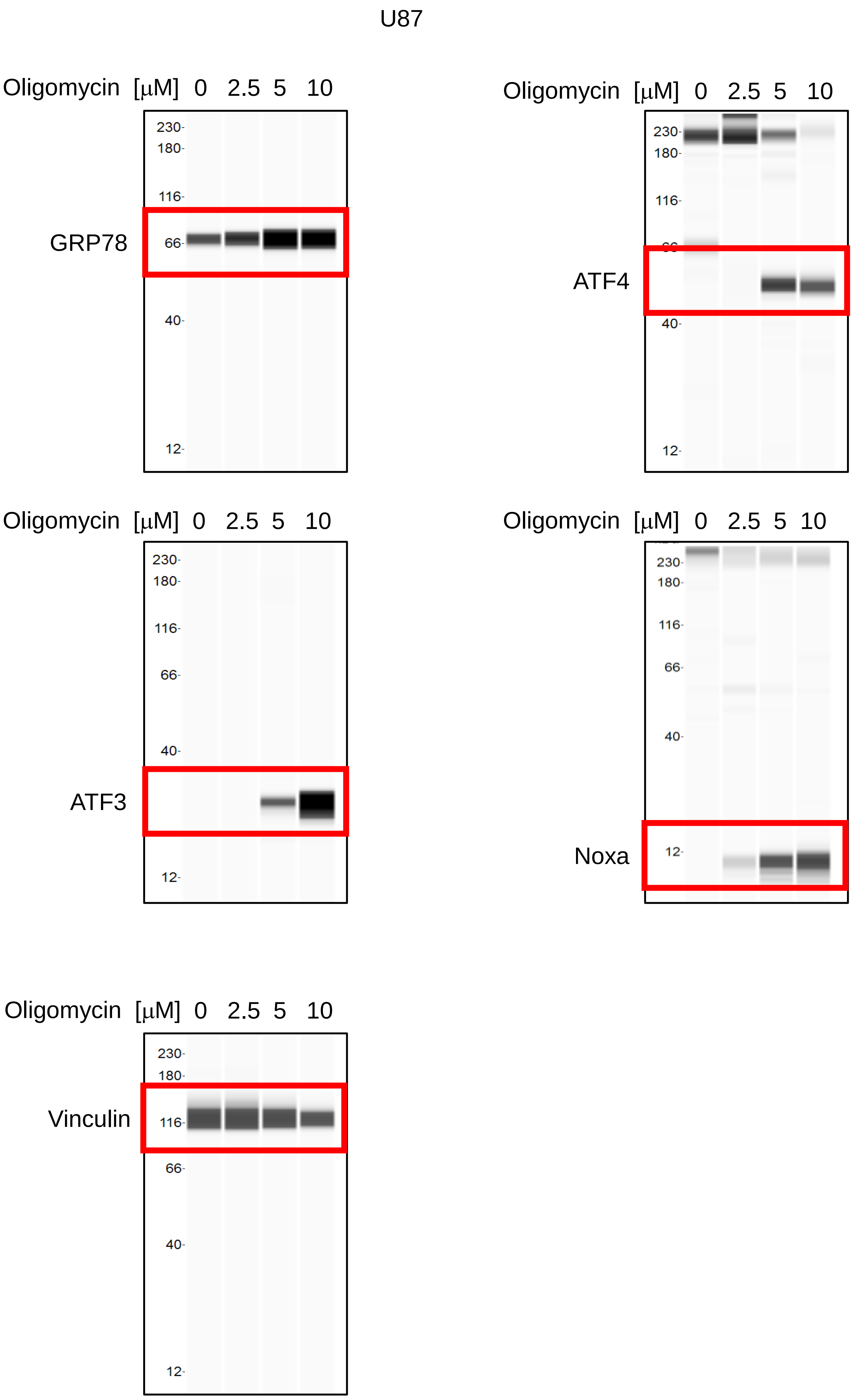

Source Data Figure S5J

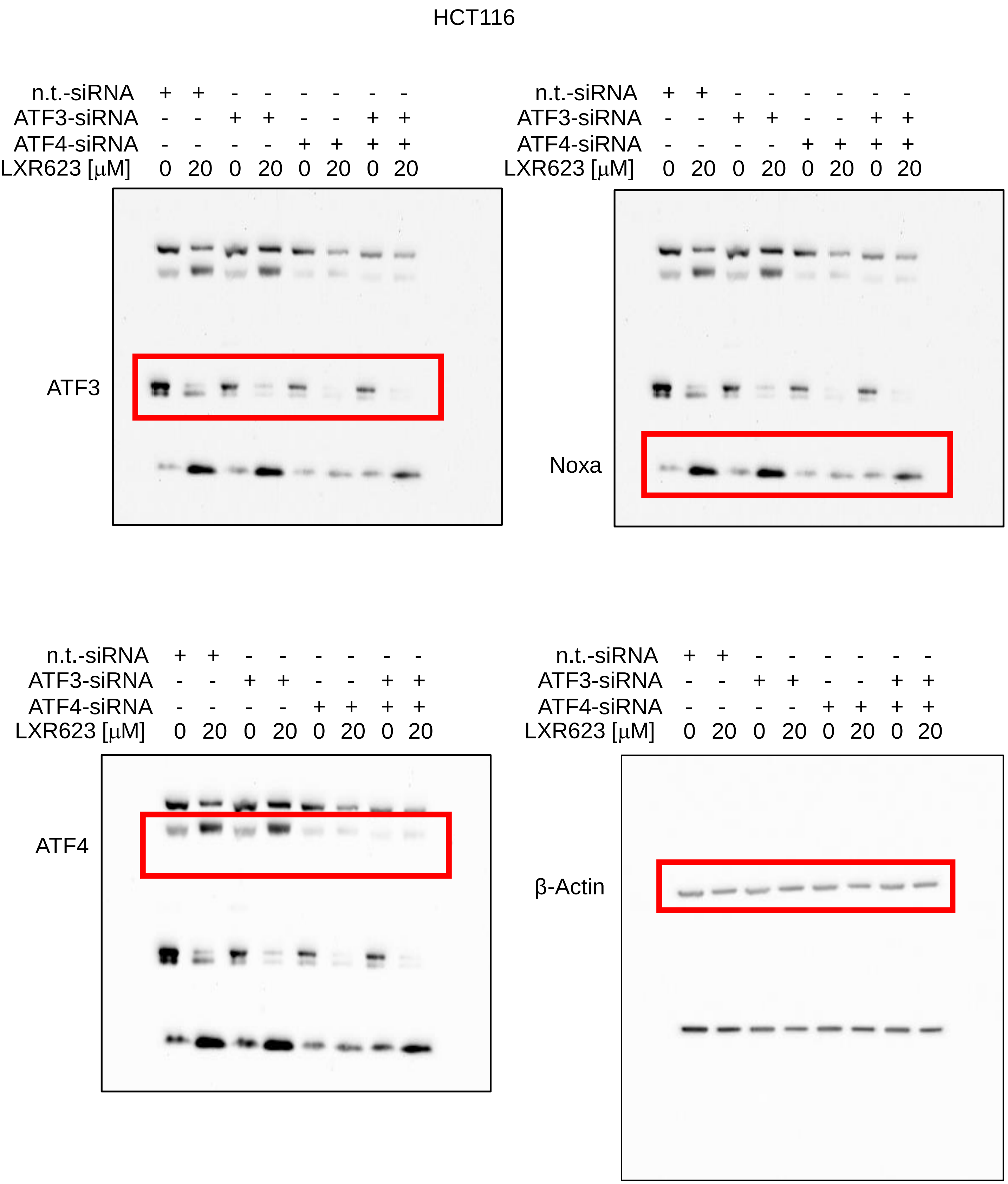

Source Data Figure S5K

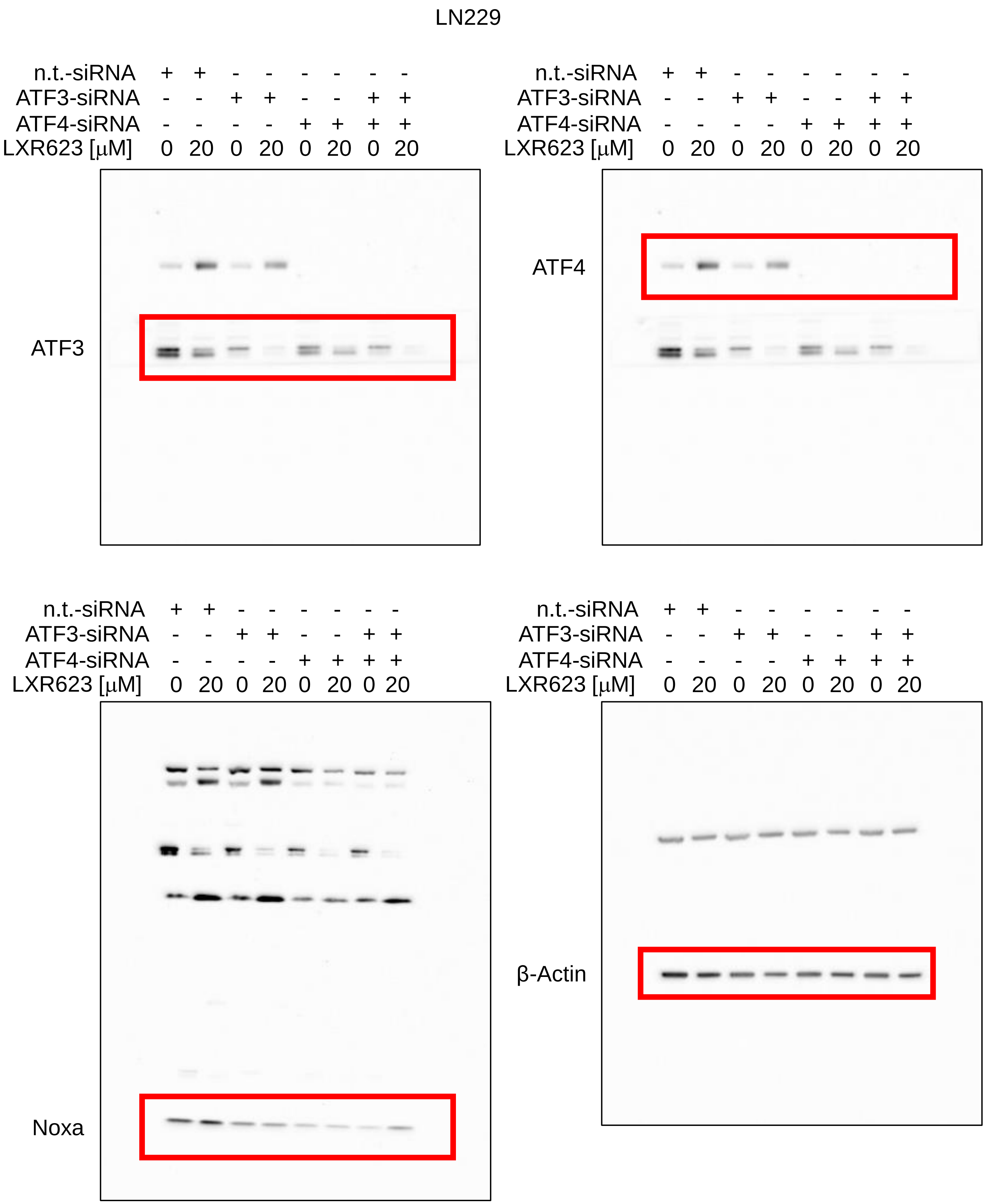

Source Data Figure S8G

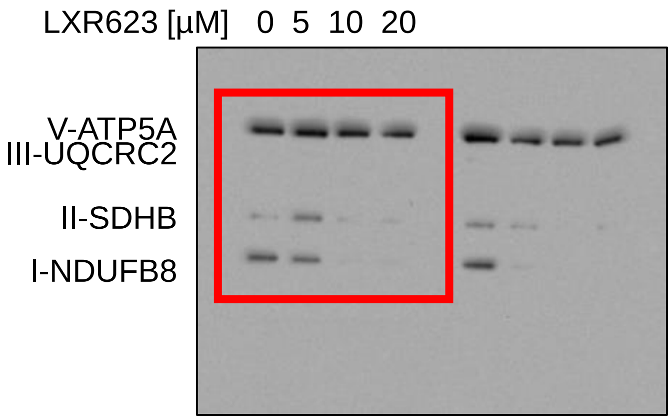

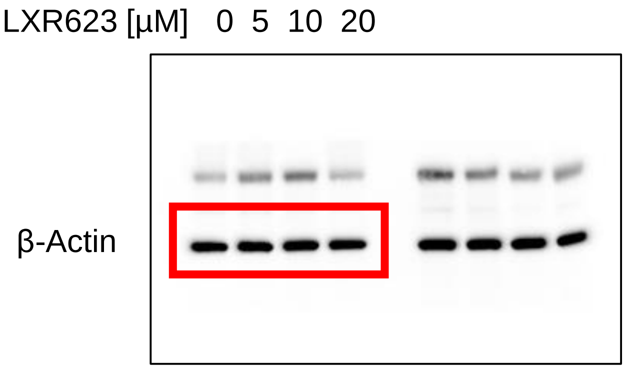

Source Data Figure S8H

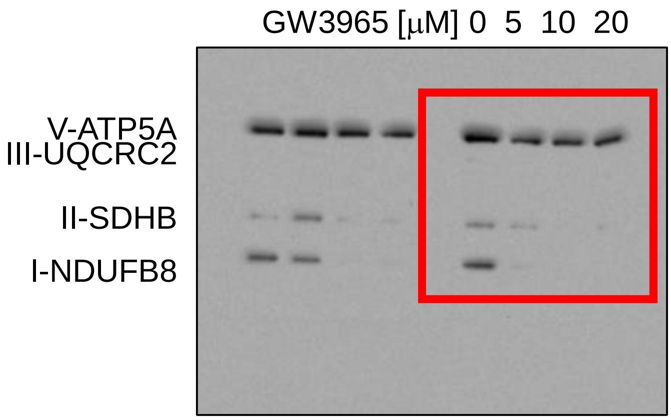

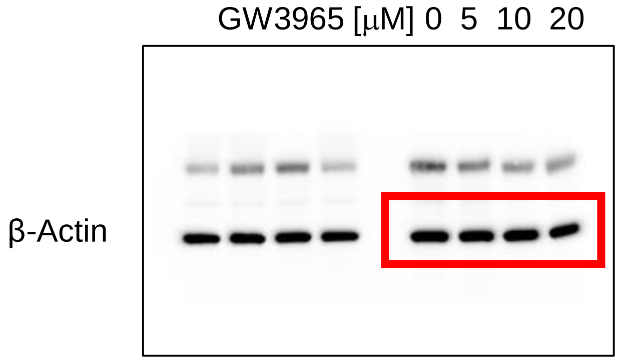

Source Data Figure S8I

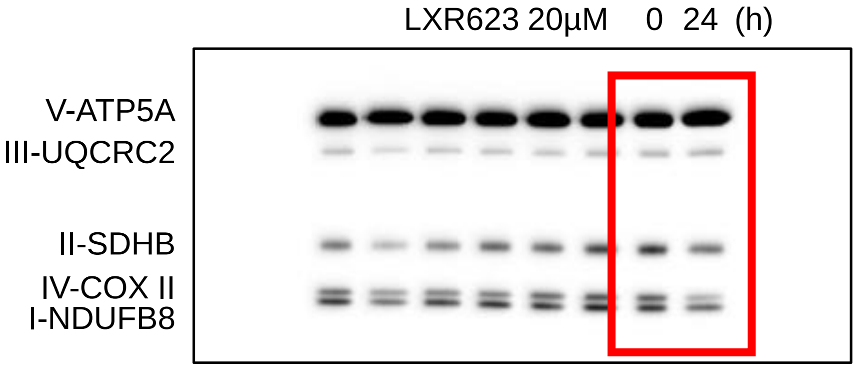

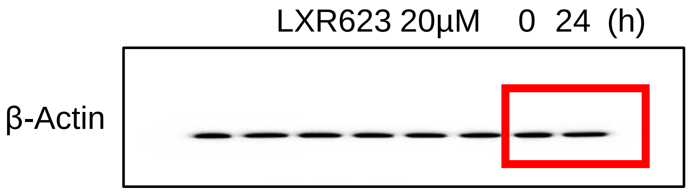

Source Data Figure S9B

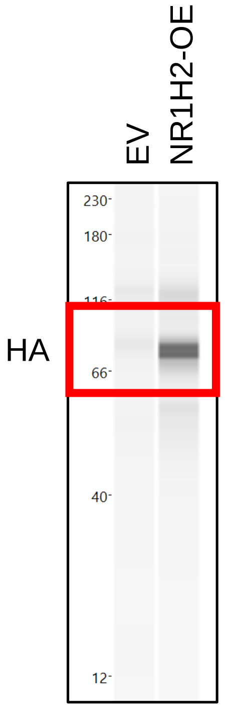

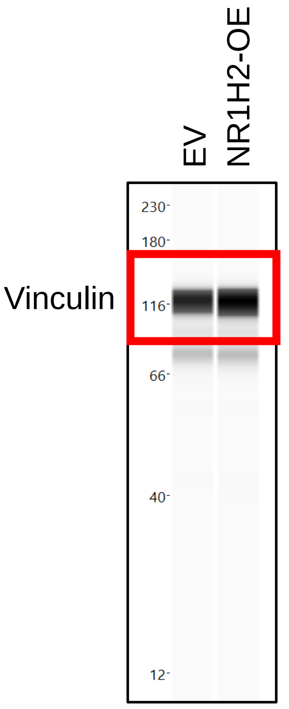

Source Data Figure S9D

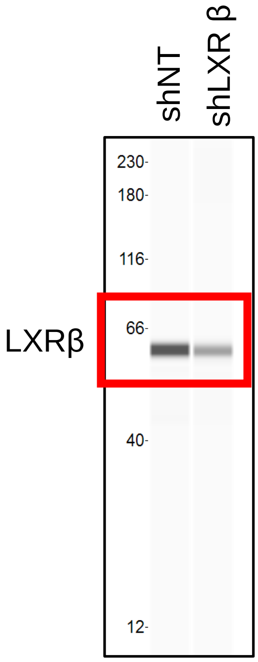

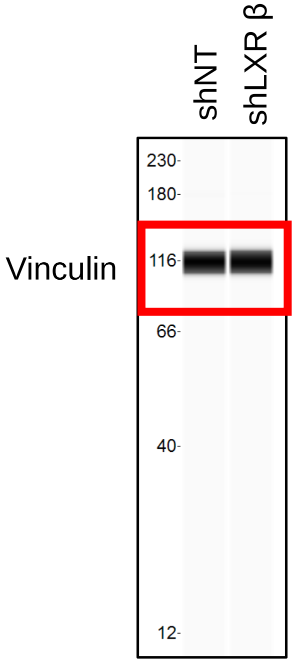

Source Data Figure S9G

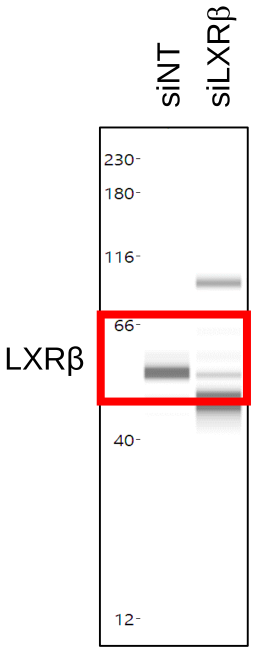

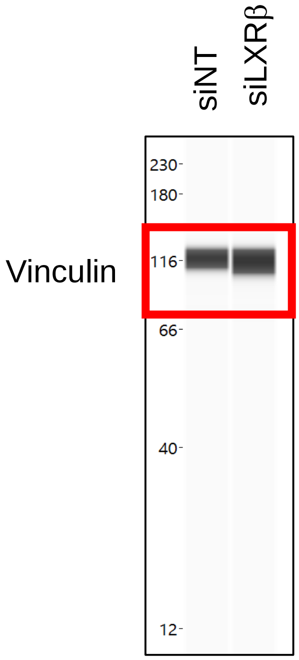

Supplement: Supplementary file 3 — Source Data for Appendix [file EMMM-11-e10769-s007.zip › Source_Data_for_Appendix_Figures.pdf]
