# Supplementary figures and images for: Activation of LXRβ inhibits tumor respiration and is synthetically lethal with Bcl‐xL inhibition
Source: EMBO Mol Med. 2019 Aug 29;11(10):e10769. doi: 10.15252/emmm.201910769 (PMC6783693; doi:10.15252/emmm.201910769)

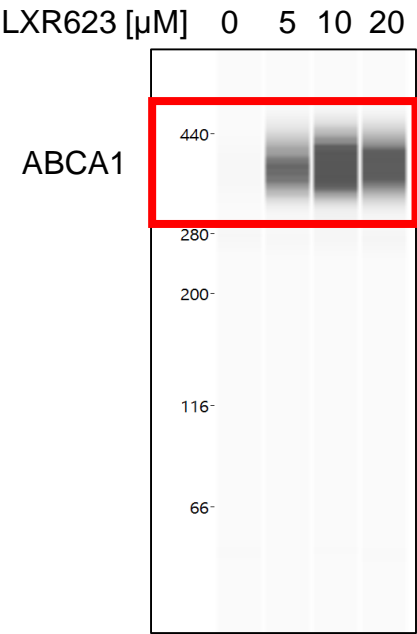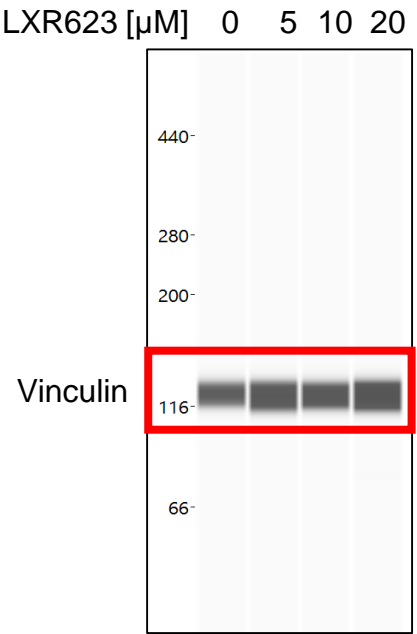

Source Data Figure 2E

LXR623 [ $\mu$ M]    0   10   0   10  
ABT263 [ $\mu$ M]    0   0   1   1

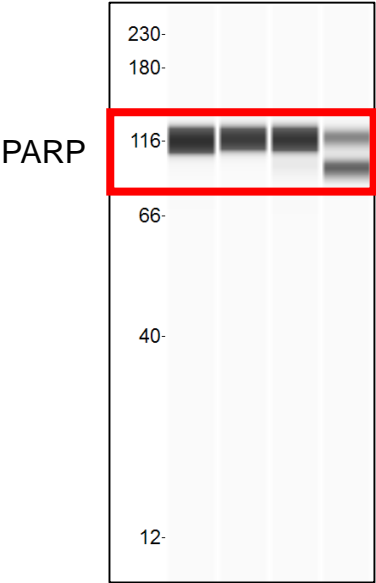

LXR623 [ $\mu$ M]    0   10   0   10  
ABT263 [ $\mu$ M]    0   0   1   1

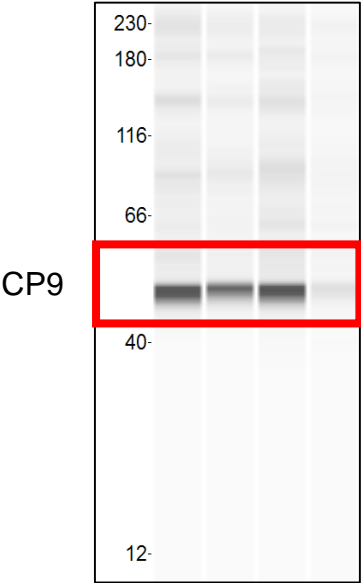

LXR623 [ $\mu$ M]    0   10   0   10  
ABT263 [ $\mu$ M]    0   0   1   1

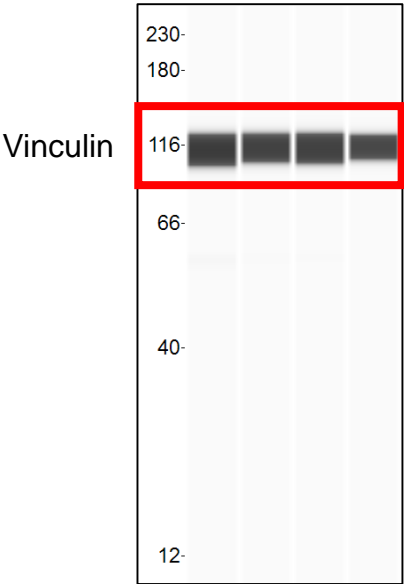

Supplement: Supplementary file 5 — Source Data for Figure 2 [file EMMM-11-e10769-s003.pdf]

LN229

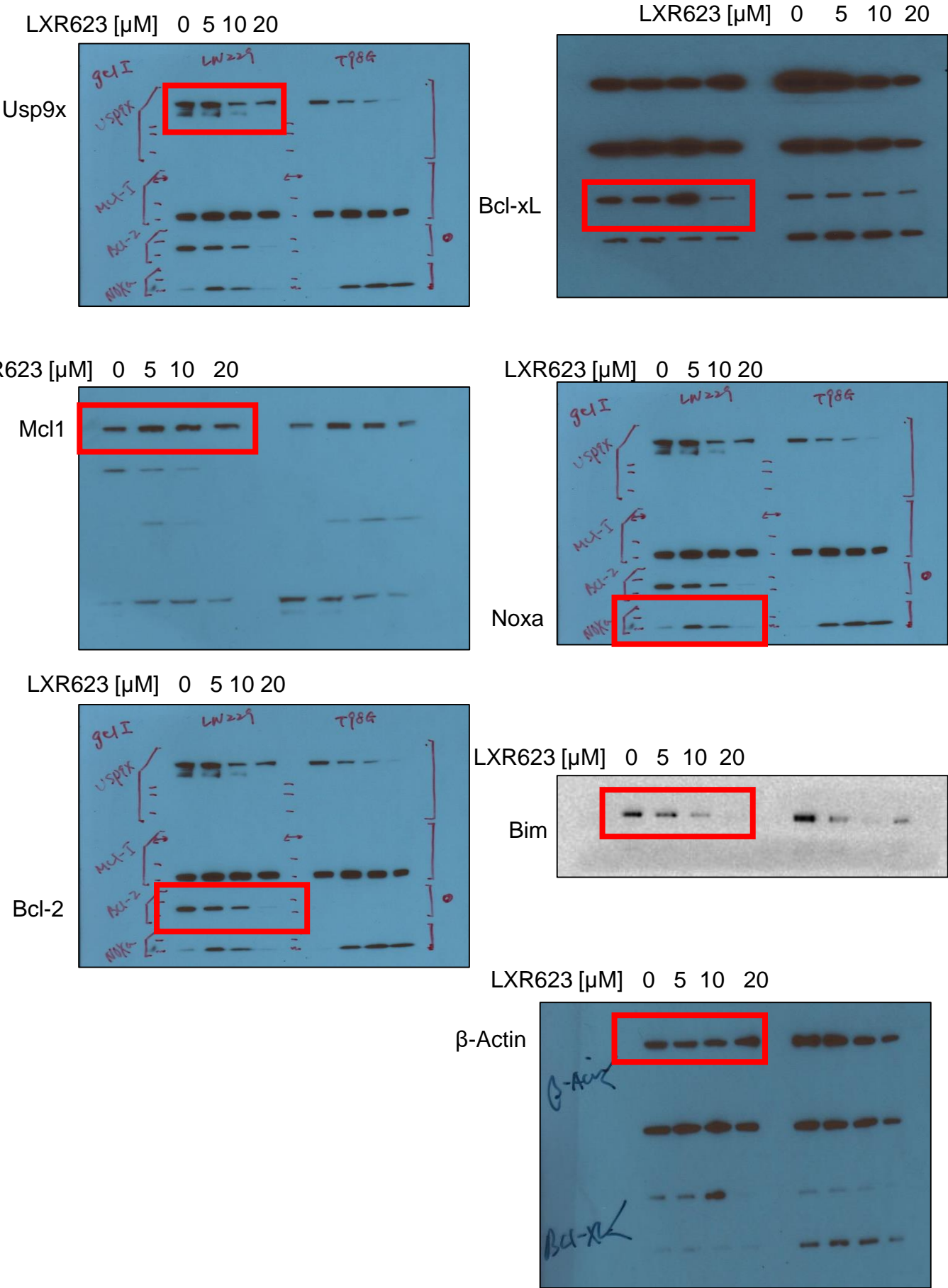

T98G

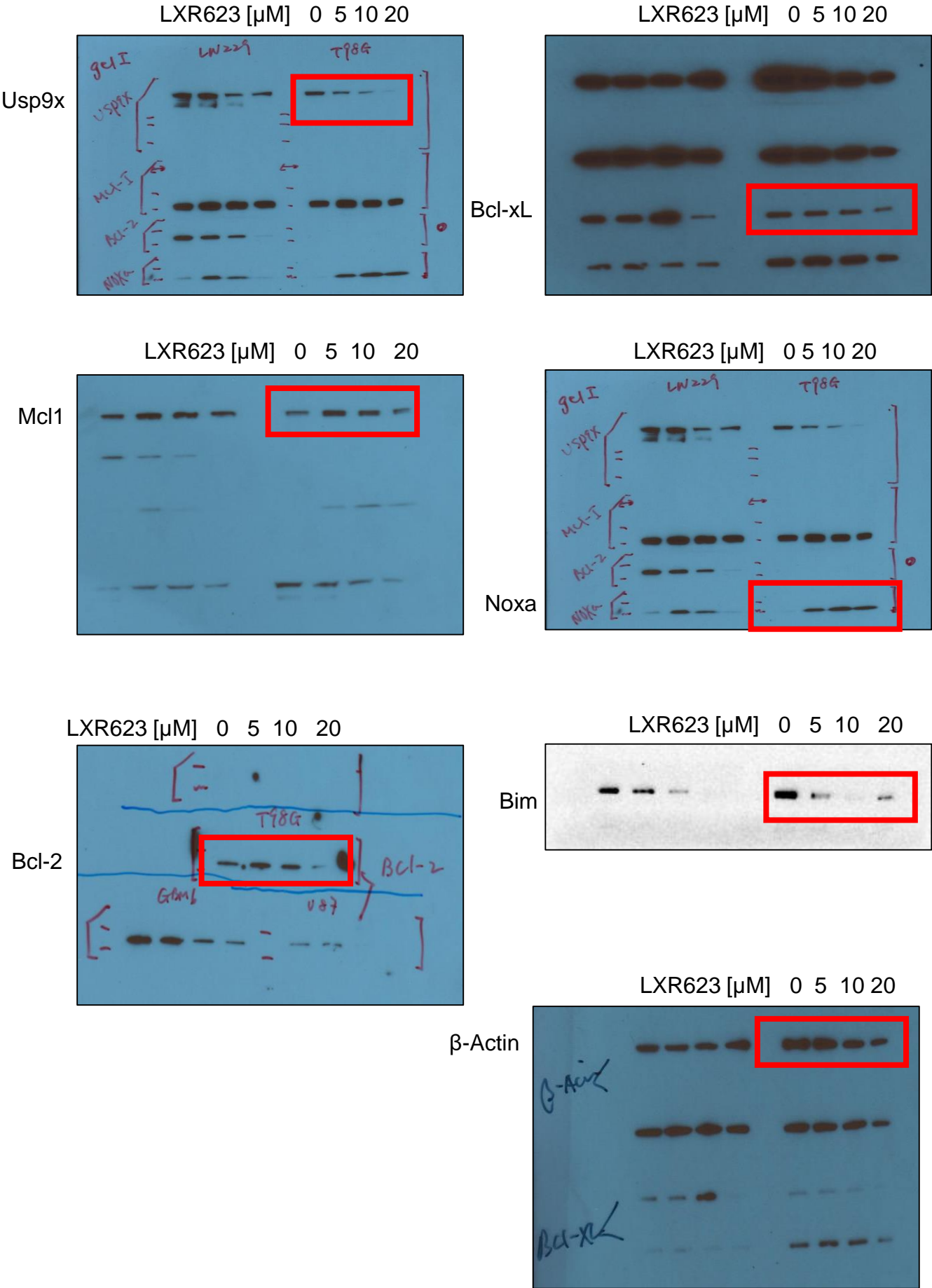

U87

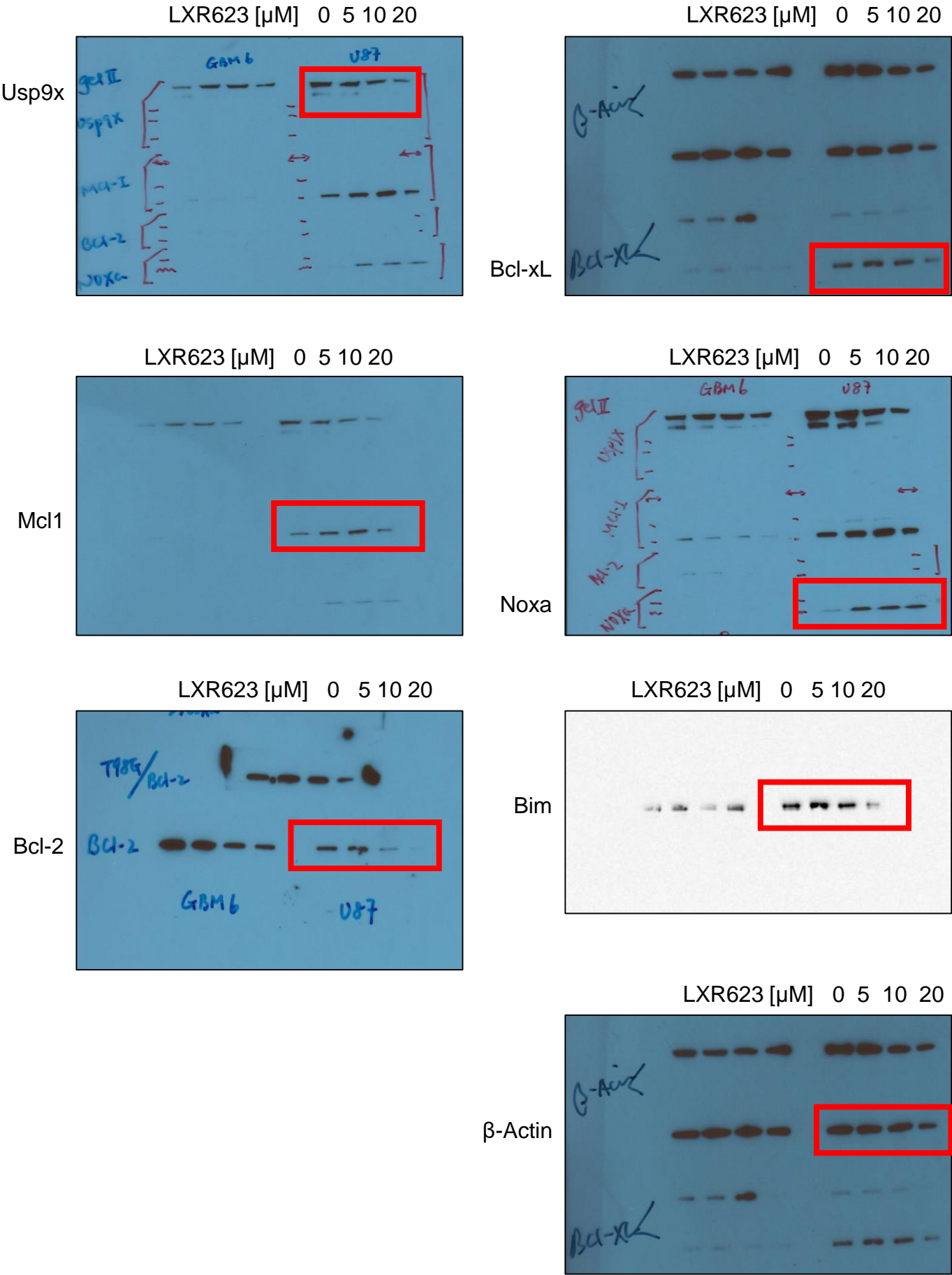

U87-EGFR-vIII

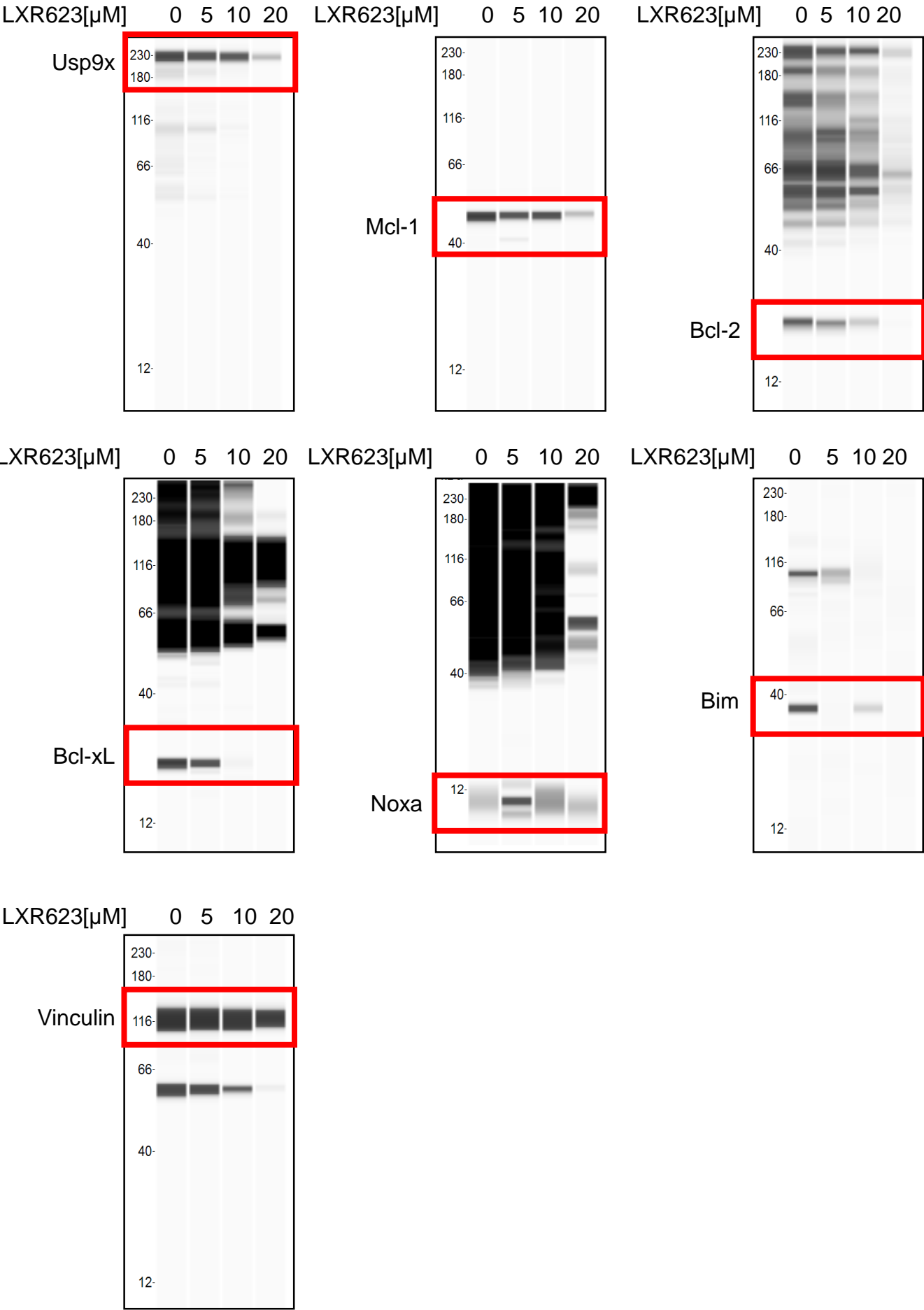

HCT116

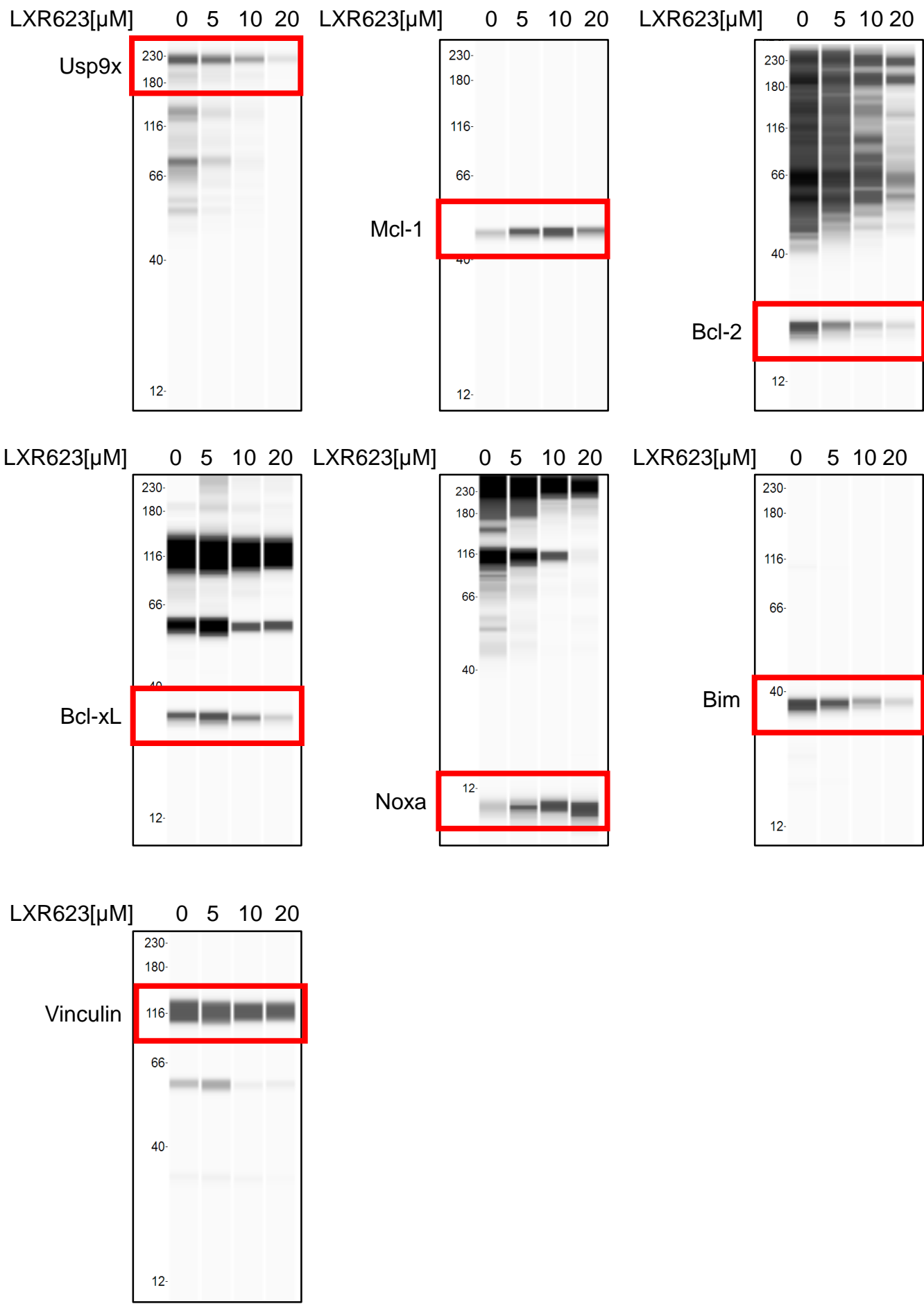

LN229

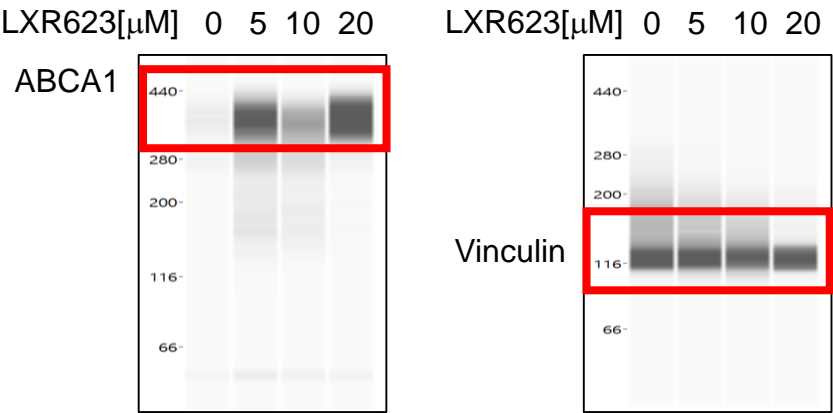

T98G

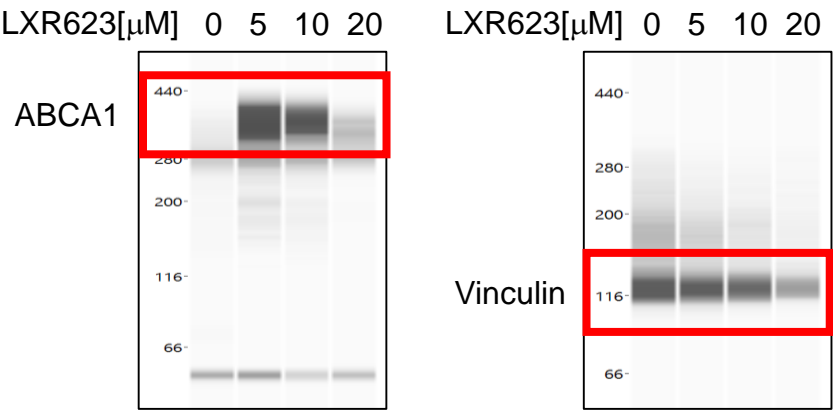

U87

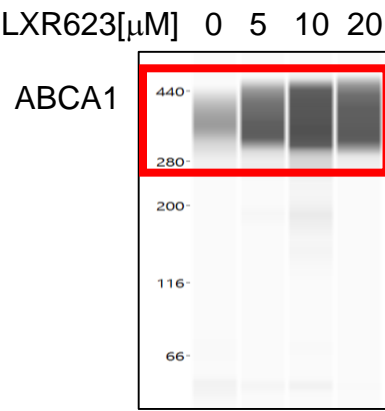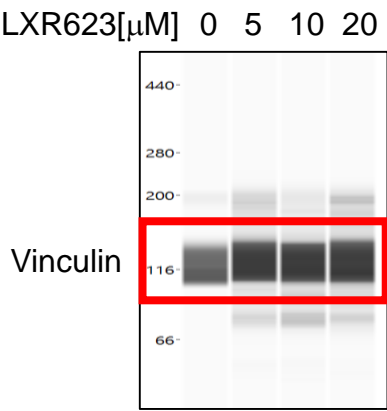

U87-EGFR-vIII

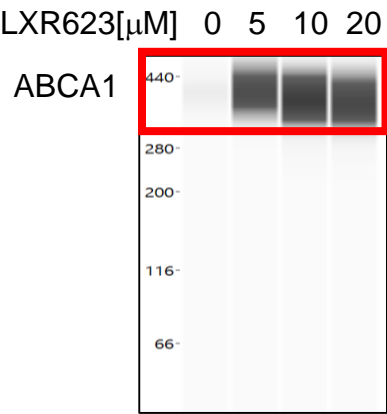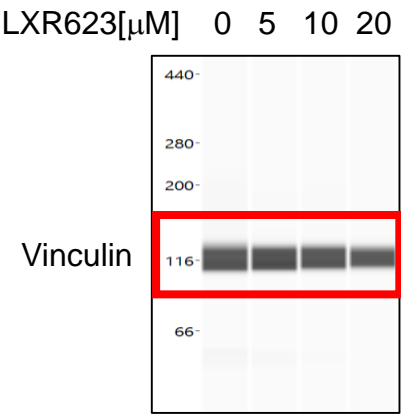

HCT116

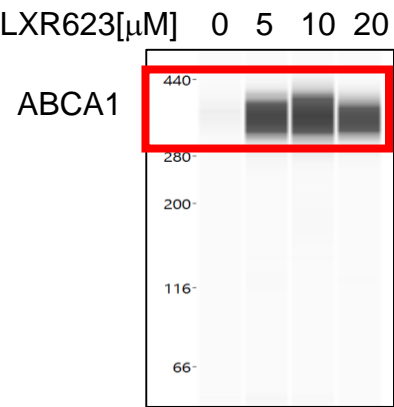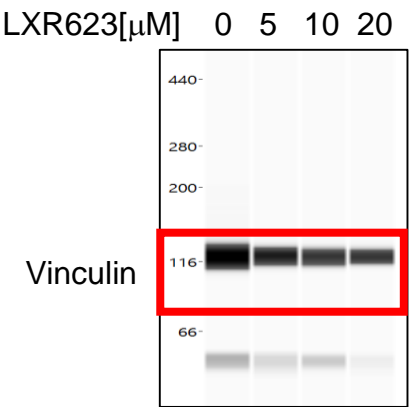

Source Data Figure 3D

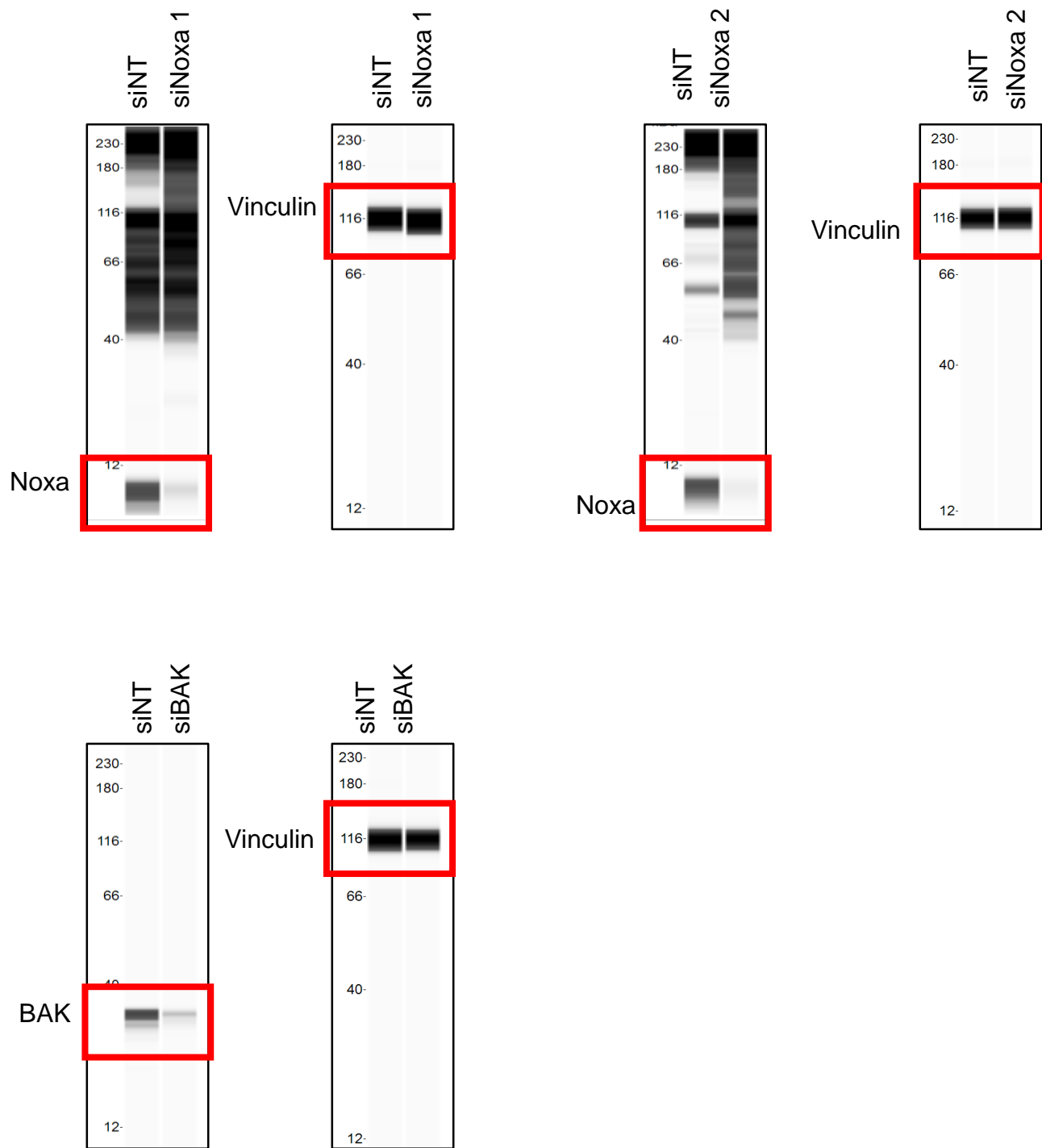

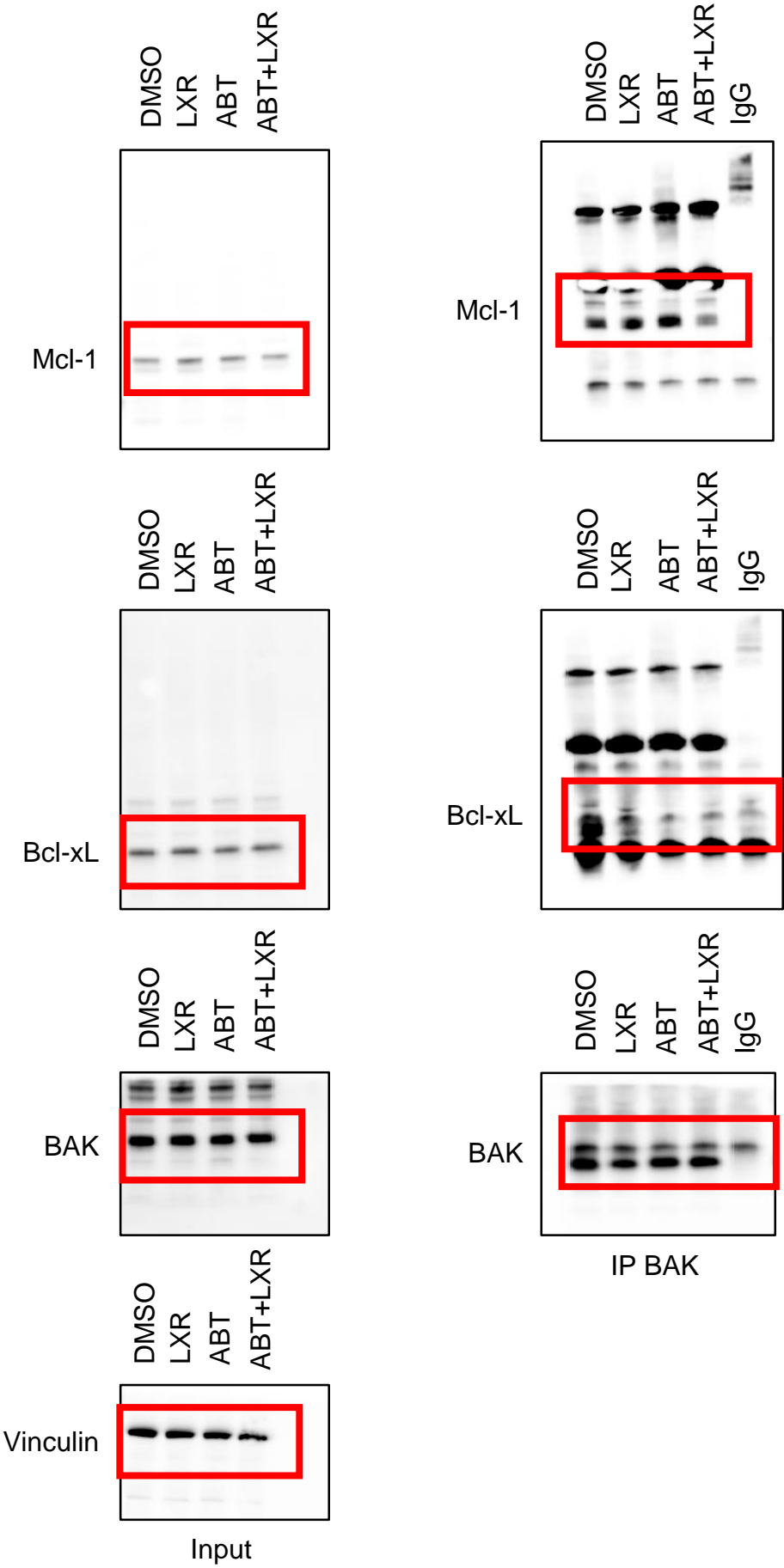

Supplement: Supplementary file 6 — Source Data for Figure 3 [file EMMM-11-e10769-s004.pdf]

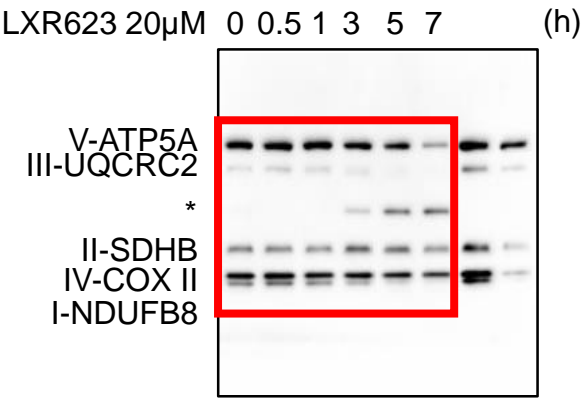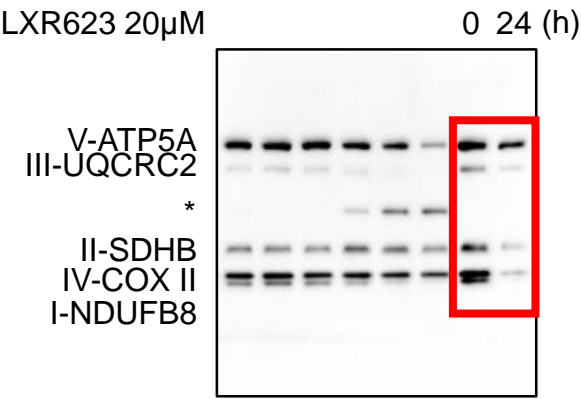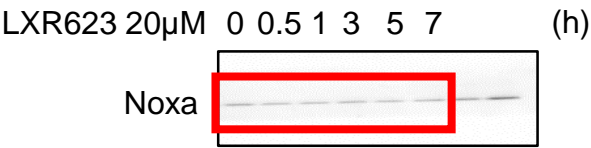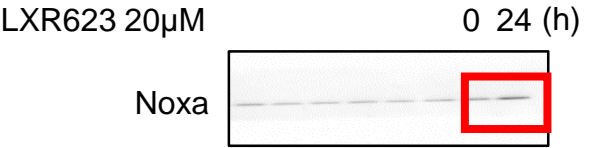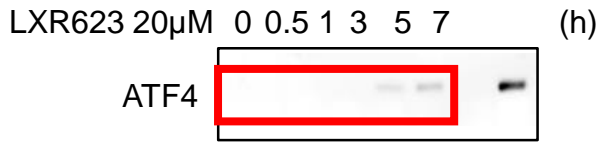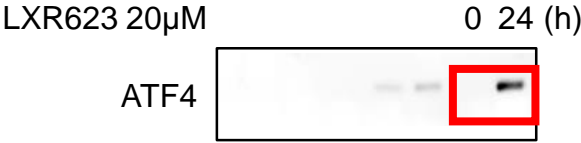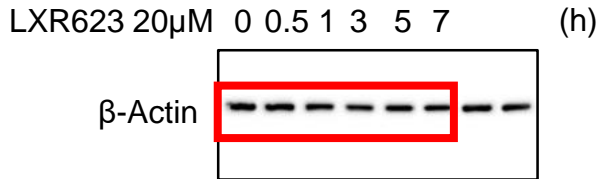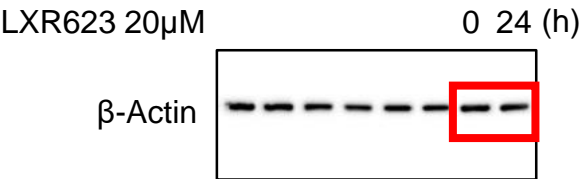

DMSO

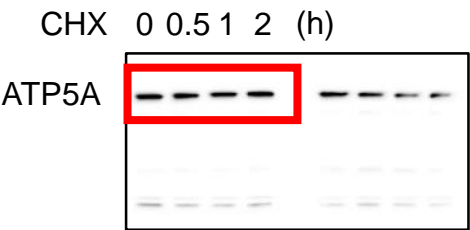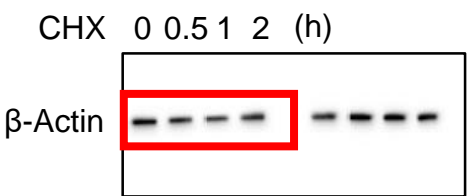

LXR623 20μM

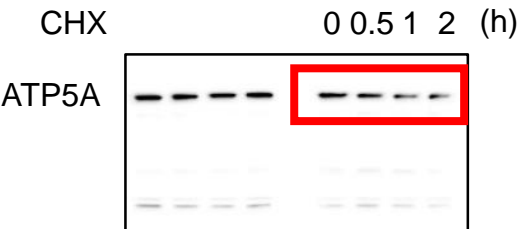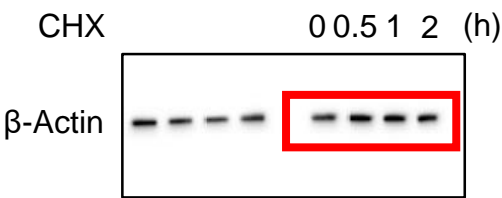

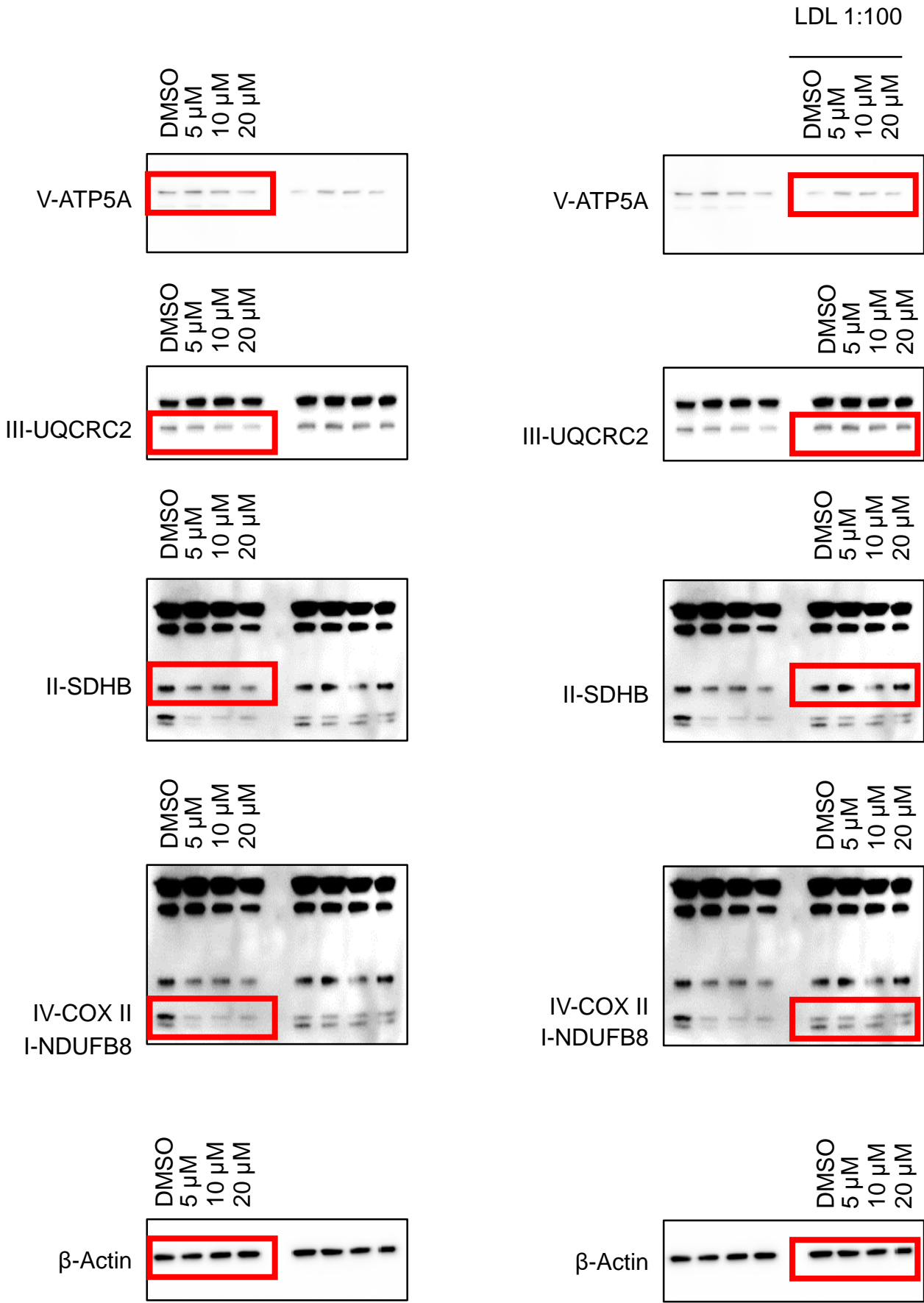

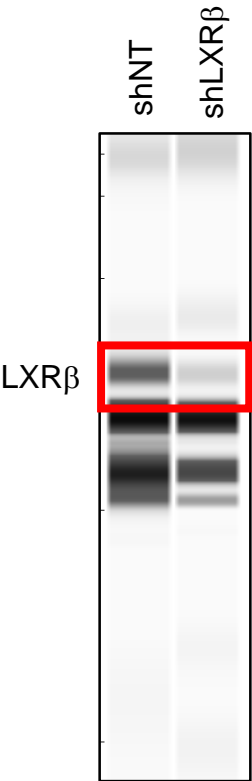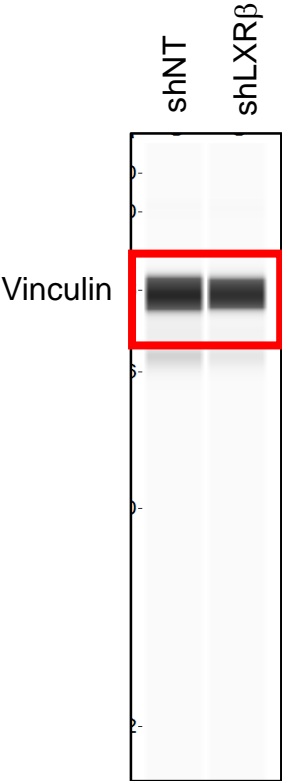

Supplement: Supplementary file 8 — Source Data for Figure 6 [file EMMM-11-e10769-s006.pdf]
