# Supplementary material for: Activation of LXRβ inhibits tumor respiration and is synthetically lethal with Bcl‐xL inhibition
Source: EMBO Mol Med. 2019 Aug 29;11(10):e10769. doi: 10.15252/emmm.201910769 (PMC6783693; doi:10.15252/emmm.201910769)

Source Data Figure 4D

7 h

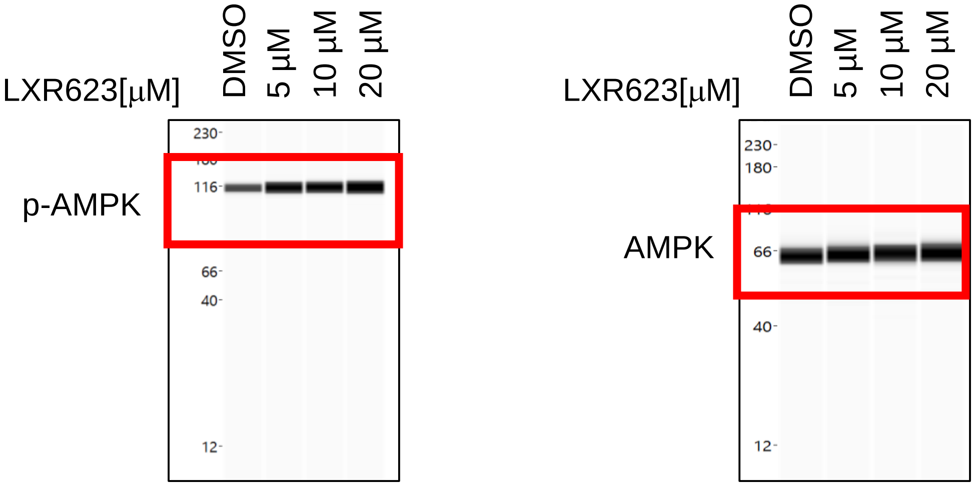

24 h

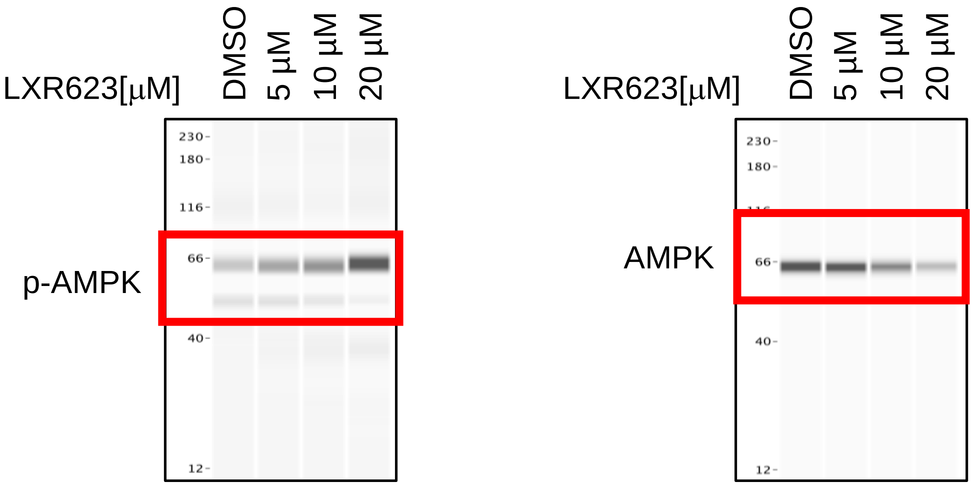

Source Data Figure 4E

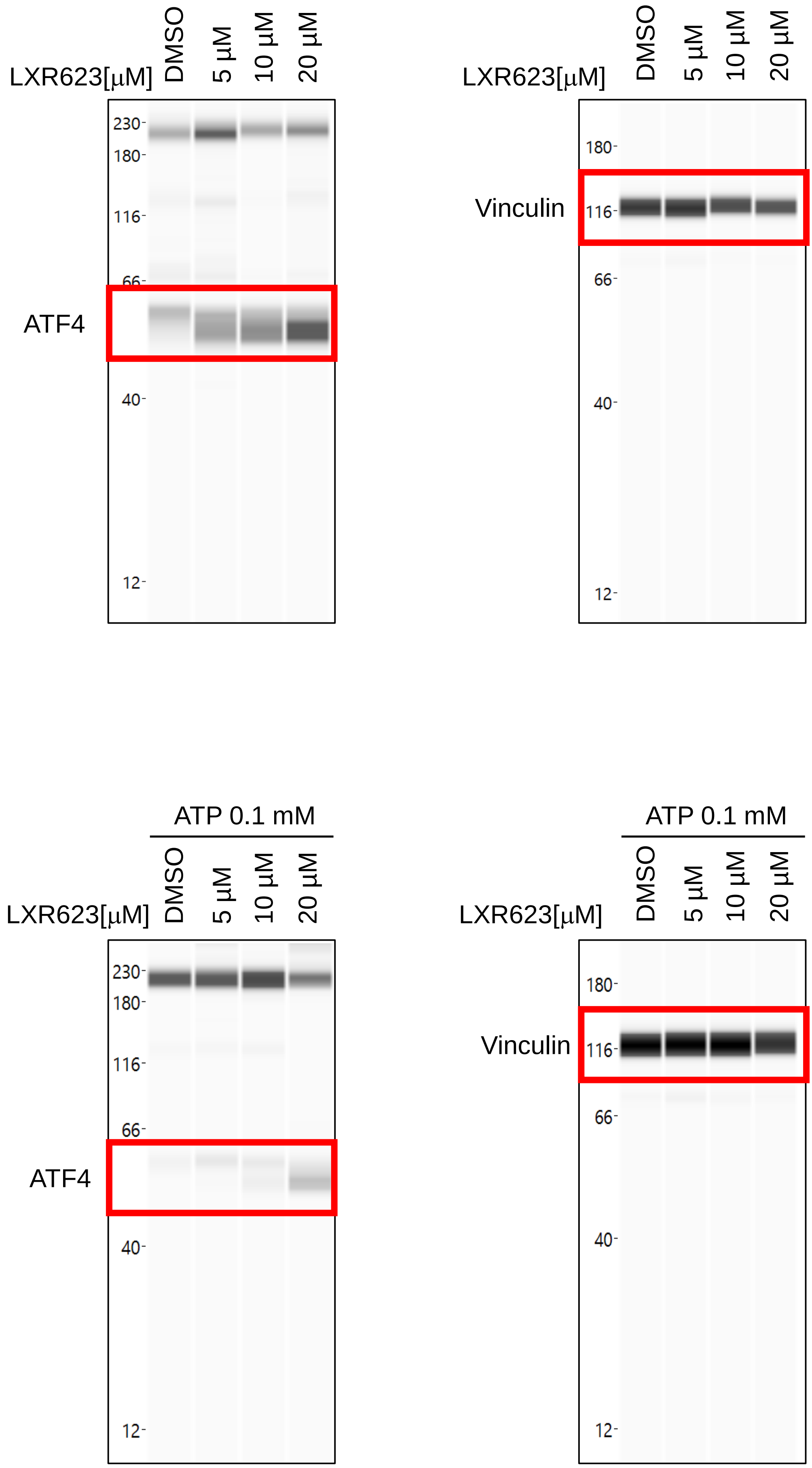

Source Data Figure 4F

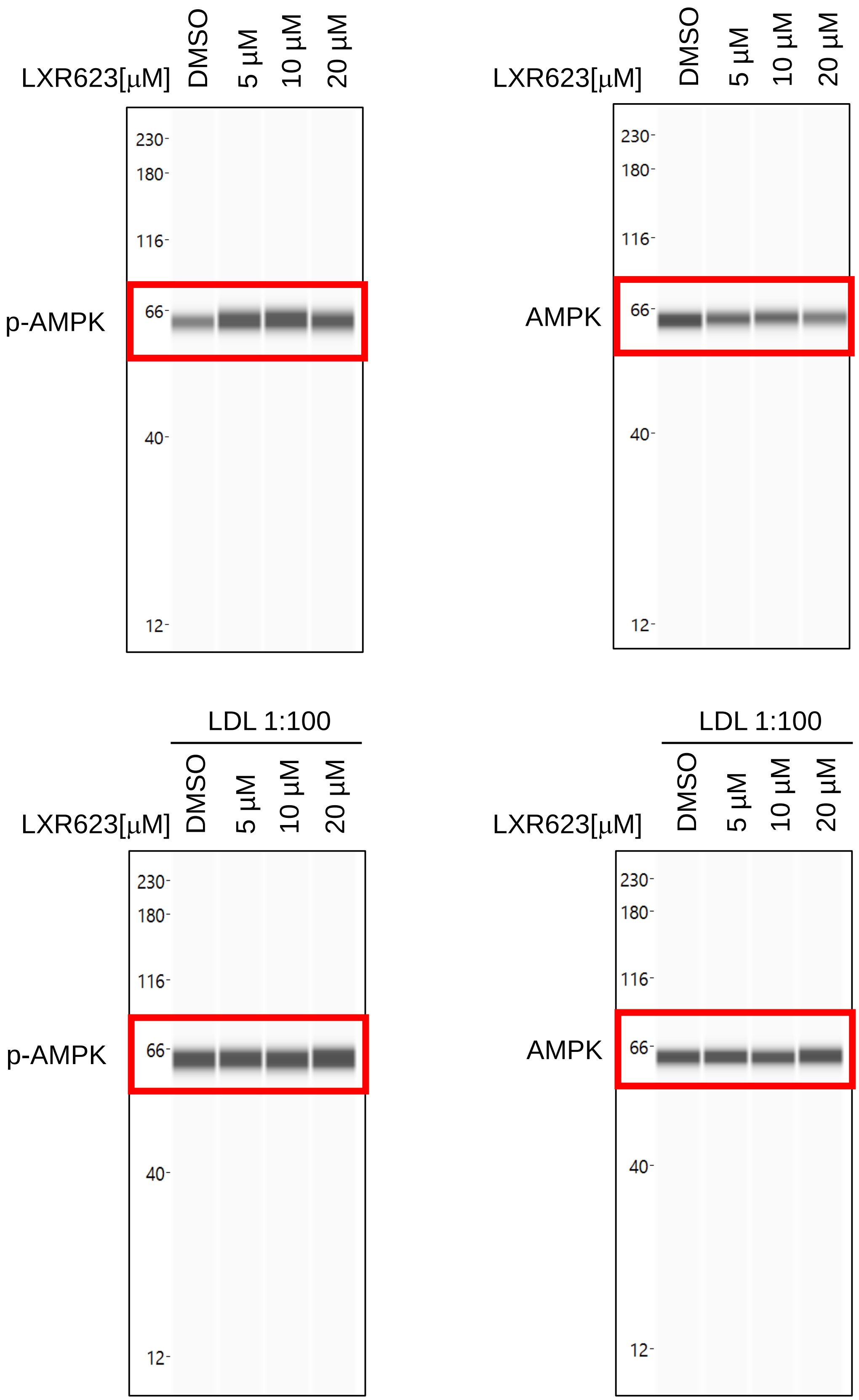

Source Data Figure 4G

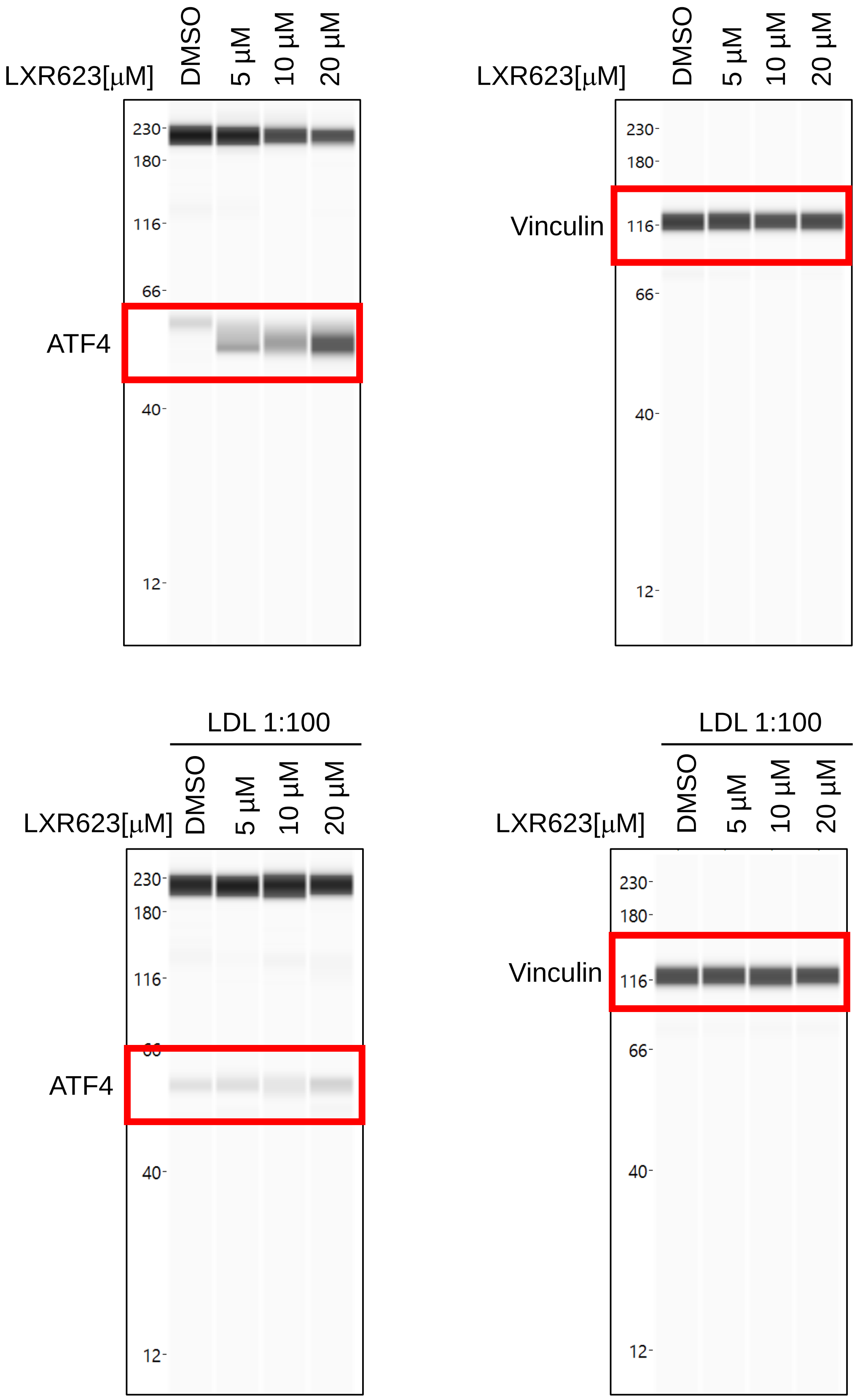

Source Data Figure 4H

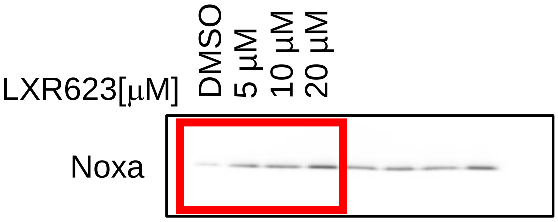

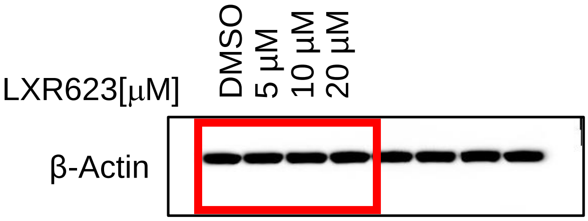

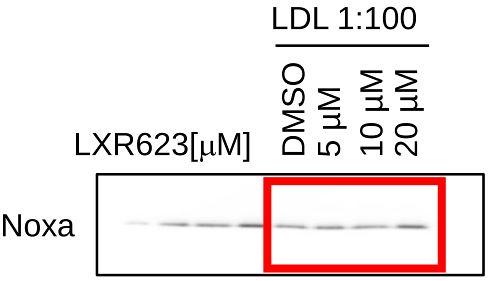

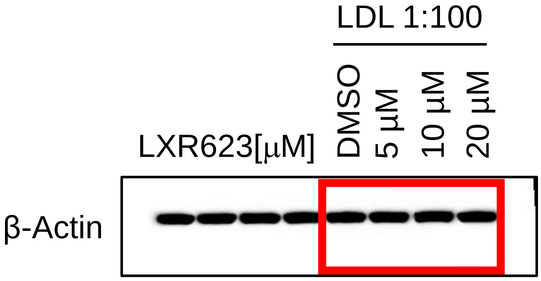

Source Data Figure 4I

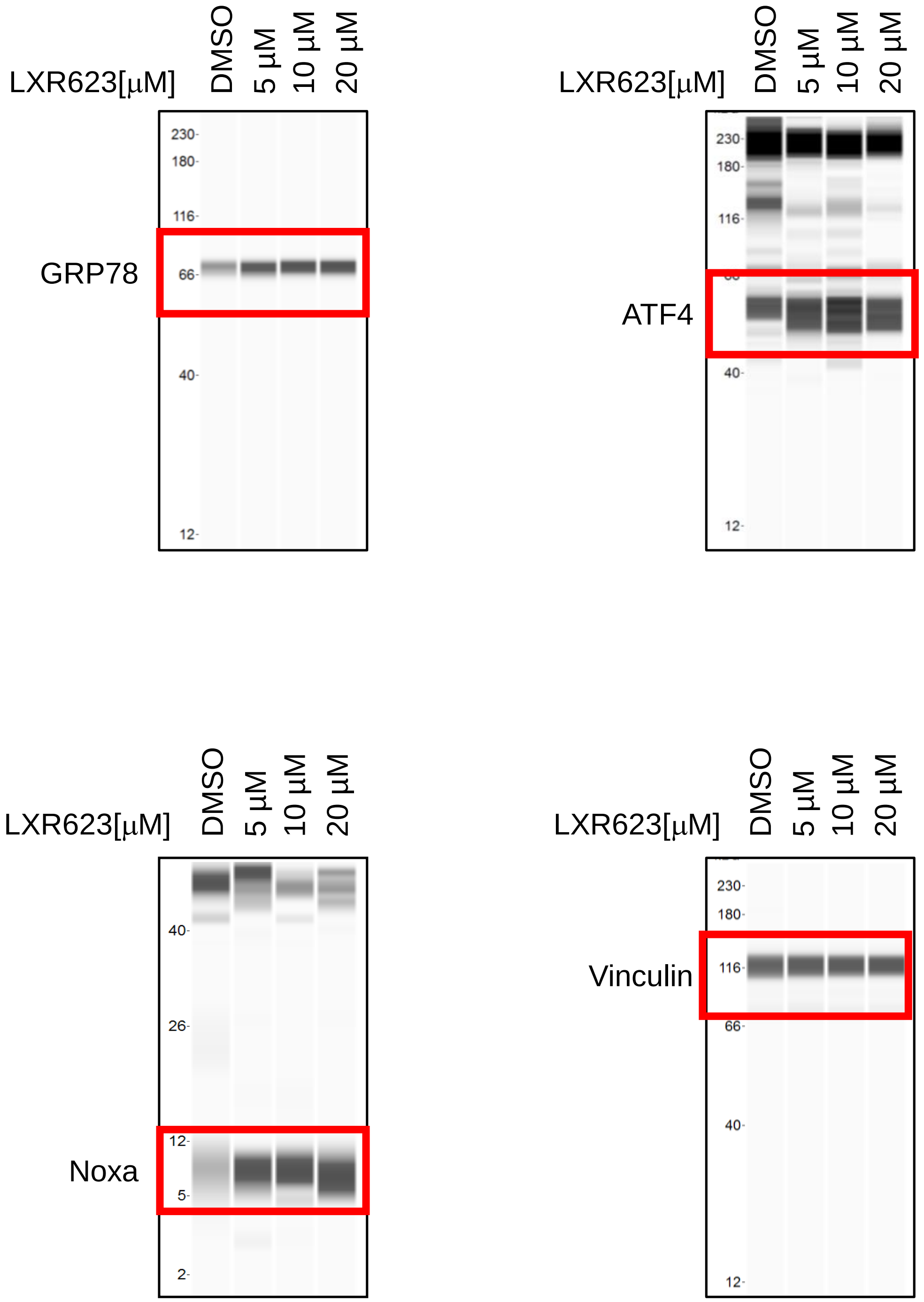

Source Data Figure 4J

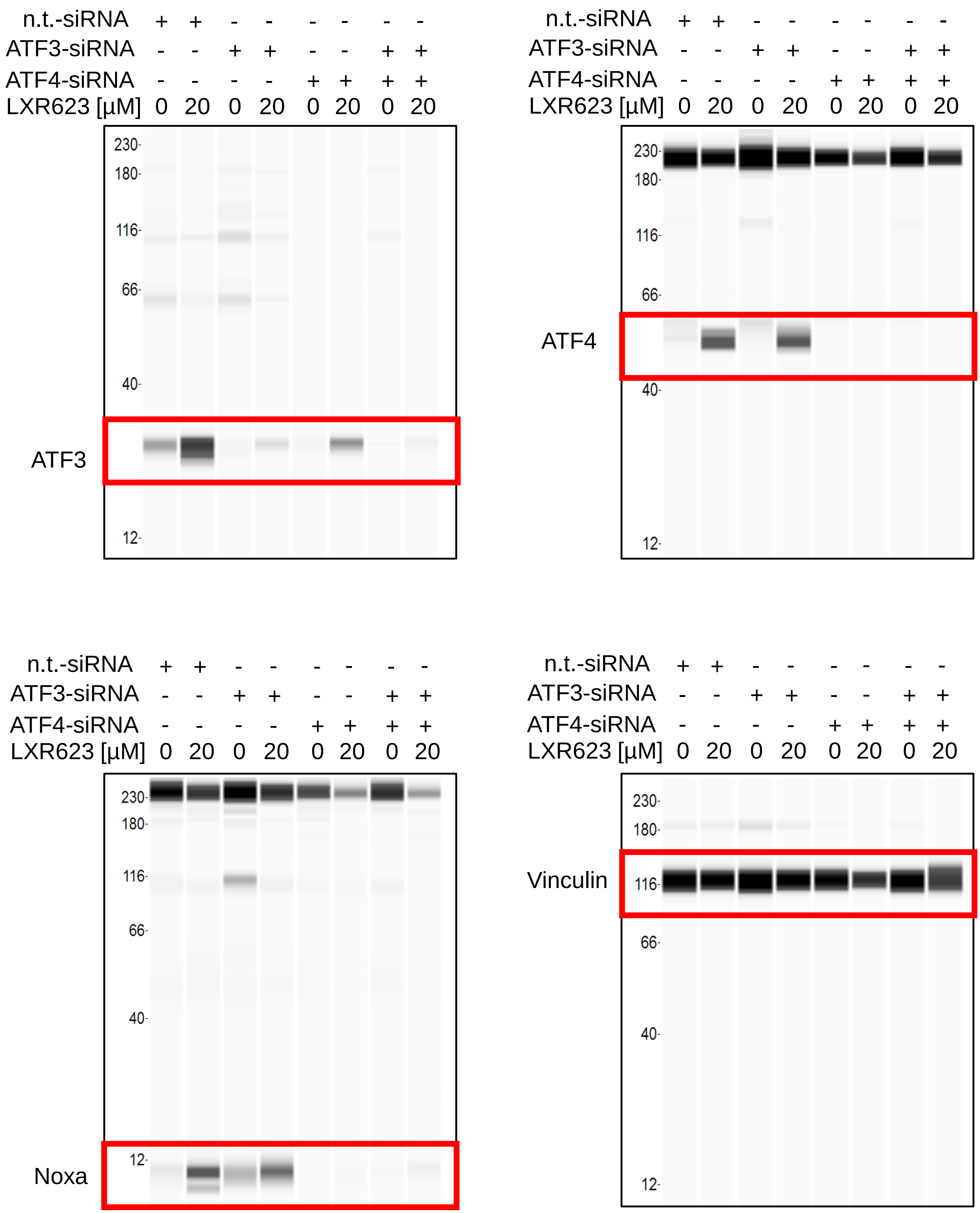

Supplement: Supplementary file 7 — Source Data for Figure 4 [file EMMM-11-e10769-s005.pdf]
